# Asymmetric spillover connectedness between clean energy markets and industrial stock markets: How uncertainties affect it

**DOI:** 10.1371/journal.pone.0316171

**Published:** 2025-03-31

**Authors:** Ailing Li, Bingmao Zhong

**Affiliations:** 1 School of Finance, Harbin University of Commerce, Harbin, China; 2 Postdoctoral Research Station of Northeast Asia Service Outsourcing Research Centre, Harbin University of Commerce, Harbin, China; Bucharest University of Economic Studies: Academia de Studii Economice din Bucuresti, ROMANIA

## Abstract

As the global climate crisis intensifies, clean energy is becoming increasingly important, and the intrinsic link between industry and energy highlights the connectedness between the industrial stock market and the clean energy market, and examining this connectedness can reveal risk spillovers between these markets. We categorise the clean energy market into hydro, wind and solar markets, and the industrial stock market into low-carbon portfolios, high-carbon portfolios and ordinary portfolios, and use the network connectedness methodology to investigate the connectedness of returns between the clean energy submarkets and the industrial stock submarkets in the time and frequency domains. The returns are categorised into positive and negative returns in order to investigate the asymmetry in the connectedness of the markets. Finally, we explore the effects of EPU, GPU, and CPU in terms of network connectedness. It is revealed that clean energy submarkets are net receivers of risk, industrial stock submarkets are risk transmitters. The hydropower market is the main risk receiver, while the low-carbon portfolio is the main risk transmitter. Risk spillovers are mainly driven by short-term spillovers and do not have persistent spillover transmission. Bad news has a greater impact on network connectedness, leading to higher levels of connectedness between markets. EPU and CPU have significant effects on network connectedness. Our findings are informative for both investors and policymakers.

## 1. Introduction

As the climate problem becomes more serious, countries must actively take various measures to mitigate and prevent further damage to the environment, of which accelerating the construction and popularisation of clean energy is an important part. Clean energy sources, including hydropower, wind power and solar power, emits virtually no greenhouse gases and produces little or no pollution, making it an important factor in reducing the burden on the environment while developing the country’s economy. As one of the world’s largest investors in the energy transition, China has been leading the way in recent years. According to Bloomberg New Energy Finance’s Energy Transition Investment Trends 2024, China’s investment in driving the energy transition could total $676 billion in 2023, accounting for 38 per cent of last year’s total global energy transition investment. To achieve the goal of “carbon neutrality” by 2060, it is anticipated that the Chinese government will maintain its robust support for green energy, which will facilitate substantial growth in the clean energy market. The rapidly growing green energy has captivated investors in China’s financial markets, who are increasingly interested in clean energy-related stocks [1]. It is difficult for public financial resources to meet the funding needs for the fast development of the green energy industry, and it is a good solution to address the lack of funds for clean energy development to encourage and guide investors to invest in green energy-related stocks [2]. In this context, it is particularly vital to follow the development of the clean energy sector in the stock market.

Industry is an essential part of a modern economy and plays a key role in a country’s development and prosperity. According to China’s National Bureau of Statistics, from 2013 to 2021, China’s industrial added value will grow at an average annual rate of 6.1%, much higher than the growth rate of other major economies in the world. In 2021, China’s industrial added value will grow by 9.6% year on year, boosting the economy by 3.1 percentage points and contributing 38.1% to the growth of China’s gross domestic product (GDP), which is an essential support for the stable running of China’s national economy. The industrial sector is not only the backbone of the real economy, but also has a key role to play in the stock market. A great majority of industrial enterprises are listed on China’s stock market. The industrial sector plays an important systemic role in the Chinese stock market and is a net risk sender in the stock market [3,4].

The relationship between green energy and the industrial sector is not complicated, as it is an upstream and downstream link in the chain, with the industrial sector needing energy for production and clean energy providing part of the industrial sector’s needs. Chinese government’s determination to be “carbon neutral” by 2060 will lead to a growing proportion of clean energy in the industrial sector, and a deeper link between clean energy and industry. In the stock market, owing to economic linkages or contagion mechanisms between sectors, there is a clear link between share price volatility of different sectors [5]. Theoretically, the connection between clean energy and industrial sectors in the market for stocks can be interpreted from two angles. In terms of economic linkages, clean energy and the industrial sector are upstream and downstream. Rising share prices of clean energy-related companies drive these companies to continuously expand clean energy, and the larger the scale, the lower the cost. Conversely, changes in the cost of clean energy affect the cost in the industrial sector. Falling production costs and rising earnings for a company are good news for the company and can lead to a rise in the company’s share price.In turn, a decline in clean energy stock prices can lead to a decline in industrial sector stocks. Changes in the industrials sector’s stock prices can also affect changes in the share prices of green energy companies. An increase in the price of industrial sector stocks encourages companies to expand production, which requires more energy, and this in turn causes clean energy stocks to rise. In terms of financial contagion, it also accounts for the price linkages between the clean energy sector and the industrial sector. Financial turbulence in one market or sector can affect other markets or sectors through channels such as investor behaviour, leading to the propagation and spread of risk from one market or sector to others, especially sectors with substantial linkages [5].

In addition, there are many different subsectors in the green energy market such as hydropower, wind power, solar power, etc., and with significant differences between the different subsectors [6]. The industrial stock market can similarly be divided into high carbon emitting industries, low carbon emitting industries and general carbon emitting industries, and the market characteristics of different carbon emitting industries also behave differently [7]. Considering that the clean energy market and the industrial share market can be divided into different submarkets, the study of the spillover relationship between submarkets is a deeper exploration of the spillover relationship between the clean energy and the industrial stock market, and the spillover relationship between specific submarkets can also provide more detailed insights for policymakers and investors. Therefore, this paper will explore the spillover relationship between the submarkets of the clean power and the industrial stock sector from the submarkets.

Owing to the development of different directions in spillover index modelling, it is possible in this paper to investigate the connectedness between clean power and industrial stock sub-markets from different perspectives. First, this paper follows Diebold and Yılmaz’s (2014) spillover model (DY spillover model) to explore the spillover effects between clean energy submarkets and industrial stocks submarkets from a time-domain perspective, which analyses not only the static connectedness between these markets, but also the dynamic risk spillovers between these markets [8]. Second, this paper considers that the results of spillover indices in different frequency bands may reflect the speed of information processing and dissemination between different markets, so this paper will adopt the Baruník and Křehlík’s (2018) approach (BK spillover model) to capture the spillover indices in different frequency bands [9]. Finally, we explore the asymmetry of spillovers by categorising returns into positive and negative returns and examining the spillover indices between market returns in different directions. The model is capable of analysing changes in spillover relationships between markets in response to the influence of positive and negative news. Furthermore, the results can be employed as a stability test.

Uncertainties are also the focus of our research. Economic, political, and climatic uncertainties have a greater impact on spillovers between financial markets [10]. Economic uncertainty refers to the uncertainty of a country’s or global economy due to various unpredictable factors. When a country or global economy is in turmoil, this uncertainty rises immeasurably and spillovers between markets soar [11]. Political uncertainty is a situation in which economic agents are unable to accurately foresee the future political environment and policy direction due to the unpredictability of political events, policy changes, election outcomes, or other political processes. Changes in political uncertainty can trigger turbulence in financial and energy markets, especially when large energy-exporting countries have tense political conflicts with other countries, and countries stockpile energy for their own energy security, which triggers abnormal volatility in the energy market [12]. Due to the prevalence of environmentalism, climate change and the concerted development of climate change-related policies by countries are also having an increasing impact on financial markets. Climate uncertainty refers to the unpredictability of future climate conditions for economic agents caused by changes in climate change and related climate policies. Climate uncertainty mainly affects energy markets and green finance markets, especially when supported by national policies, the impact of climate uncertainty on spillovers between markets is significant [13]. In order to explore the effects of economic uncertainty, political uncertainty, and climate uncertainty on spillovers between clean energy and industrial stock markets, this paper develops a regression model that includes these three types of uncertainty.

Although, there is a lot of prior literature exploring the relationship between the energy market and the industrial stock market, there is little literature that divides the energy market and the industrial stock market into many sub-markets and then explores the risk spillover relationship between them. Analysing spillovers from a sub-market perspective not only validates the findings of the previous literature, but also provides more insight into which of the sub-markets has the greatest impact on the spillover network. This is quite important for both financial market managers and investors. The gap between this paper and the previous literature lies in the fact that the market under study is divided into self-markets, and the asymmetric spillovers between markets are uniquely examined in both the time and frequency domains, and the impact of uncertainties on spillovers is explored.

There are five main contributions to the research in this paper. Firstly, the main research object of this paper is clean energy and industrial stock market, although there is a large amount of literature studying the spillover effect between clean energy and other financial markets, but there are few studies linking clean energy and industrial stocks, so the research in this paper will make up for the lack of this direction. Second, this paper uses a combination of DY and BK spillover index models to analyse the risk spillover effects between clean energy and industrial stock markets in a multi-dimensional and multi-angle way. Third, this paper divides the clean energy market into three sub-markets, namely, hydro, wind and solar, and divides the industrial stock market into three markets according to carbon emissions, so as to study the spillover effects between the respective sub-markets of the clean energy and industrial stock markets, and to explore the relationship between the clean energy and the industrial stock markets from a more subtle perspective. Fourth, this paper classifies market returns into positive and negative returns and investigates the asymmetry of risk spillovers between markets under good news and bad news shocks, expanding the study of asymmetric spillovers. Finally, this paper incorporates uncertainty into the study of risk spillovers and identifies the impact of various types of uncertainty on risk spillovers.

The key findings of this paper are that the clean energy market is a receiver of risk and the industrial equity market is a sender of risk for the vast majority of the sample period; the risk spillover index fluctuates dynamically over time, spiking rapidly when a huge event occurs; and analysed in terms of the frequency bands, spillovers between markets are mainly driven by short-term spillovers. Considering the various markets in the segmentation, it can be observed that hydro energy is the largest recipient of risk and fluctuates dramatically during large events; the low-carbon industrial stock market has been a transmitter of risk over the sample period, and both the high-carbon and general industrial stock markets have been recipients of risk for periods of time. In terms of the asymmetry of spillovers, risk spillovers between markets are stronger with bad news shocks than with good news shocks. In addition, economic policy uncertainty and climate policy uncertainty have a significant effect on spillovers between clean energy and industrial stock markets, and geopolitical uncertainty has a non-significant effect.

The remainder of the paper is structured as follows. Section 2 is a literature review. Section 3 presents the methodology and data. Section 4 presents the empirical results and discussion. Finally, Section 5 concludes the paper.

## 2. Literature review

### 2.1 Spillover effects in clean energy markets and industrial stock markets

#### 2.1.1 Spillover effects in clean energy markets.

In light of the mounting urgency of environmental concerns, green energy has emerged as a pivotal area of inquiry for scholars in recent years. In particular, the spillover linkage between clean power and other financial markets has been the subject of extensive study by numerous scholars [2,14,15]. The identification of the spillover relationship between clean energy and other financial markets facilitates the formation of more efficient portfolios for investors and contributes to the advancement of green financial markets [16]. Pham (2019) found for the first time that there is heterogeneity in the spillover linkage between traditional financial markets and clean power subsectors and argued that policies should take into account the characteristics of different subsectors of the clean energy industry [17]. Zhang et al. (2023) showed that there is a spillover relationship between green energy, electricity and traditional energy markets, with the correlation being strong at extreme quartiles [18]. Spillover shocks to financial markets from clean energy in the context of epidemics have likewise been studied by scholars, concluding that epidemics make contagion between markets stronger [19]. Chen et al. (2022) compared the spillover relationship between clean energy, fossil energy and metals markets and found that the clean energy market changed from a spillover recipient to a spillover exporter after the Paris Agreement was signed [20]. Karkowska and Urjasz (2023) examined the risk spillover relationship between clean energy, dirty energy, and the stock market and concluded that clean energy is one of the few assets that can be used as a safe haven [21].

#### 2.1.2 Spillover effects in industrial stock markets.

Industrial stock markets have also been the main subject of academic research, usually as part of the stock market, analysing spillovers between industrial stock sector and other financial markets [22–24]. Xu et al. (2022) investigated spillovers between the stock market and the carbon market, finding that industrial stocks are risk receivers in most cases [25]. Wu et al. (2022) also explored the spillover relationship between the industrial sector and the carbon emissions market and came to a different conclusion, concluding that the industrial sector was an information transmitter for carbon emissions trading [26]. Ouyang and Shaw (2024) formed a risk spillover network of the top ten sectors of the Chinese share market and found that systemically important sectors such as industrials need more attention [27]. Nekhili et al. (2024) examined the spillover relationship of higher order moments across sectors in the European stock market and found that industry is the largest source of systemic risk [28]. Several lines of evidence suggest that the industrial sector is the largest contributor and recipient of spillovers in the system [29]. However, there is little literature that further disaggregates the industrial sector, especially by the level of carbon emissions. Zhang and Xu (2023) classified Chinese industrial stocks into clean, dirty and ordinary portfolios, and examined the spillover effects of the three portfolios with respect to oil shocks [7].

There are a number of studies in the existing literature on spillovers in clean energy markets and industrial stock markets, and it is generally agreed that these two markets have strong spillover relationships with other financial markets. This paper links the clean energy market and the industrial stock market, examining in particular the risk spillover relationship between their sub-markets.

### 2.2 Impact of uncertainties on financial markets

Uncertainty can affect financial markets in terms of the option value of investments, risk premiums, and potential bonuses [30]. At the same time, uncertainties are heterogeneous in their effects on financial markets [31]. The effects of economic uncertainty, political uncertainty and climate uncertainty on financial markets are analysed next.

#### 2.2.1 Impact of economic uncertainty on financial markets.

At the micro level, an increase in economic uncertainty causes confusion among investors as well as financial institutions, which prevents them from making optimal choices, resulting in inefficiencies in the financial markets [32]. Economic uncertainty likewise has a great impact on listed companies. Rising economic uncertainty causes companies to hold a more cautious attitude towards investment, and cash holdings rise as uncertainty rises [33]. Zhou and Chen (2024) found that economic uncertainty growth is associated with a decline in collaborative research and development among firms, which hinders innovation and growth [34]. From the perspective of the financial market as a whole, a rise in economic uncertainty may cause turbulence in the financial market, both in developed and developing countries. Mo et al. (2024) used the TENET methodology to specifically examine the risk spillover of economic uncertainty on different stock markets and found that economic uncertainty in the US plays a central role in stock market returns [35]. Through the study of Kayani et al. (2024), the effect of economic uncertainty on the stock market is significant for the BRICS countries [36]. The ‘policy-driven’ nature of the Chinese stock market makes it particularly affected by economic uncertainty, so many scholars have explored the relationship between economic uncertainty and the Chinese stock market. Wang et al. (2020) emphasised the existence of asymmetric spillover effects between the Chinese stock market and EPU [37]. Xia et al. (2020) highlighted that economic uncertainty has special predictive significance for the Chinese stock market [38]. In addition to exploring the relationship between economic uncertainty and the stock market, many other scholars have discussed the relationship between EPU and financial markets such as energy market, commodity market, carbon market, etc. [39,40].Al-Shboul et al. (2023) studied the relationship between cryptocurrencies and economic policy uncertainty, and find that economic policy uncertainty has a certain negative impact on aggregate spillovers [41].Dai and Zhu (2023) used real volatility, skewness and kurtosis as risk proxies to examine the spillover relationship between different markets and find that economic policy uncertainty is the centre of risk for extreme downside kurtosis shocks [42].

#### 2.2.2 Impact of political uncertainty on financial markets.

Political risks can directly or indirectly have a significant impact on financial markets, and political risks such as wars, terrorism, and interstate conflicts that can lead to geopolitical tensions can have a particularly dramatic impact on financial markets [43]. Mohammed et al. (2023) used the example of the Russian-Ukrainian conflict as a contingent geopolitical event, and shows that the Russian-Ukrainian conflict had a significant impact on the returns of the energy stock market [44]. The occurrence of political events causes greater disruptions to the production and exports of crude oil exporting countries, which in turn leads to production cuts in oil-producing countries as well as a decrease in the scale of crude oil exports and an increase in crude oil prices [45]. The occurrence of political events can cause economic instability, which to a certain extent affects the consumption and demand of crude oil [46]. For investors, the occurrence of political events can cause investors to panic about the increase of crude oil prices, which pushes up the price of crude oil futures out of the consideration of reducing losses [47]. In addition, the occurrence of political events can also enable investors to create hedging demand, which in turn leads to price volatility in the crude oil market [47]. The fact that crude oil is the most important raw material for the industrial sector makes it inherent that fluctuations in the price of crude oil will lead to changes in the cost of production for companies, which in turn will cause volatility in the stock market [48]. Li et al. (2024) particularly emphasised that rising political uncertainty will lead to enhanced risk spillovers from the crude oil market to the energy stock market [32]. Zheng et al. (2023) pointed out that when political events occur, political uncertainty acts as a risk transmitter, channelling risk to other markets [49].

#### 2.2.3 Impact of climate uncertainty on financial markets.

As the climate problem is getting worse, countries are jointly or on their own introducing many policies to protect the natural environment, which have the potential to cause economic or financial shocks of varying degrees [50]. Policies for a low-carbon transition may cause the share prices of fossil fuel companies to fall, and the share prices of companies utilising clean energy are likely to rise. Xu et al. (2023) pointed out that climate uncertainty arising from the response to climate change may lead to equity risk, and investors who are unable to judge what direction the policy will take will reduce their long-term equity investments [51]. In addition, when major climate risks are encountered, executive orders imposed to minimise losses may also affect financial markets [52]. Lu Wei (2023) emphasised the heterogeneity of the impact of climate uncertainty on different sectors of the stock market, while surfacing that climate uncertainty has a good effect in predicting stock market volatility [53]. Ji (2024) incorporated several financial markets including the stock market in the systematic analysis at the same time and found that climate uncertainty has significant spillover effects on different financial markets [54]. Ding et al. (2022) argued that climate change has a long-term causality in the transmission of shocks between the carbon market and the fossil energy market [19]. Wang et al. (2023) discussed the relationship between climate uncertainty and risk spillovers between three financial markets, pointing out that climate uncertainty is a receiver of risk [13].

In summary, uncertainty arising from economic, political, and climate changes can lead to changes in the real economy, as well as investment concerns of investors, which in turn affect financial market volatility. Uncertainty arising from the response to economic and climate policy uncertainty and the occurrence of geopolitical events can easily trigger economic and financial market turbulence in this way. It is therefore necessary for this paper to incorporate all three types of uncertainty into the framework at the same time, and to explore how uncertainty will affect spillovers from clean energy and equity markets.

### 2.3 Risk spillover methodology

In addition, as scholars delve deeper into the study of financial markets, the methods of studying price linkages and volatility between different markets have been updated. For example, ARIMA model, cointegration test, Granger causality test, vector autoregression (VAR), and vector error correction model (VECM) are used to identify yield correlation and mean spillover [55–59]. In terms of volatility, GARCH family models, stochastic volatility (SV) models, and extreme risk measures (e.g., VaR, CVaR, ES) are used to analyse volatility aggregation and spillover effects [60,61]. Dynamic conditional correlation (DCC-GARCH) model, BEKK model, risk spillover (CoVaR, MES) model and systematic risk (SRISK) model are applied to investigate the dynamic characteristics of volatility [62,63]. Mamman et al. (2023) based on a panel GARCH model to explore the BRICS countries’ stock market response to economic policy uncertainty [64]. Gong et al. (2021) used a TVP-VAR-SV model to investigate the strength of time-varying spillovers between carbon and fossil energy markets [60].

Since the emergence of the DY model, more and more scholars have argued that this model has unique advantages in constructing risk networks. Ali et al. (2024) used the TVP-VAR model to identify mean spillovers and their volatility and use the DY model to construct a spillover network between green cryptocurrencies and the G7 stock market [65]. Ali et al. (2024) focused on the renewable cryptocurrencies and the GCC stock market in the context of a and concluded that they have a significant spillover relationship [66].Tan et al. (2020) used the DY spillover model to explore the risk spillover relationship between carbon markets, energy markets and financial markets [67]. Traditional econometric methods are unable to explore the spillover relationship between markets in different frequency ranges, and the BK spillover model fills this gap by capturing not only the overall spillover effect, but also the spillover effect in different frequency bands [19]. Li et al. (2023) used the BK model and found that the spillover effect between energy, metal and clean energy markets in different frequency bands has a obvious differences, with long-term spillovers dominating [16]. Ding rt al. (2021) explored risk spillovers between oil, gold and foreign exchange markets based on the BK model and found that short-term spillovers are always larger than long-term spillovers [68].

In summary, it can be seen that there are numerous methodologies for identifying spillovers and all of them have their own unique advantages. In this paper, we wish to investigate the risk spillover relationship between the clean energy market and the industrial stock market, so we decided to use the TVP-VAR model to obtain observations without losses and the DY and BK spillover network models to construct the connectedness network.

### 2.4 Research gap statement

Most of the existing studies revolve around the spillover relationships between clean energy markets and stock markets, clean energy markets and traditional energy markets, clean energy markets and power markets, etc., but there is little literature analysing the relationship between clean energy and financial markets from a segmented industry perspective, especially the lack of studies on the spillover relationship between clean energy markets in segmented industries and industrial stock markets classified by carbon emissions. In addition, while there is a large body of literature in the existing research on identifying financial market spillovers, research on asymmetric spillovers between clean energy and industrial stock markets has not yet been conducted, especially in terms of the impact of different types of uncertainty on spillover effects. Moreover, the existing literature lacks consideration of the frequency aspects of the spillover relationship between clean energy and industrial stock markets. It is important to take the frequency band of spillovers into account because only by knowing the frequency band in which spillovers occur can we understand whether spillover shocks occur in the long run or in the short run. While economic uncertainty has been described enough in uncertainty studies, the impact of geopolitical uncertainty and climate policy uncertainty on financial markets has been less well documented, and with the globalisation of the economy and the rising awareness of environmental protection, the impact of these two types of uncertainty on the economy and finance has become more pronounced, and it is therefore necessary to explore these uncertainties.

## 3. Methodology and data

### 3.1 Methodology

In order to explore asymmetric spillovers between green energy markets and industrial stock markets in terms of time-varying and frequency bands, we employ the economic model used by Chatziantoniou et al. (2023) [22]. The model consists of the TVP-VAR connectedness framework of Koop and Korobilis (2014) and the frequency connectedness approach of Baruník and Křehlík (2018) [9,69]. The TVP-VAR model, with no loss of observations as well as no restriction on the rolling window, is a suitable model for identifying risk spillovers between markets [59]. The TVP-VAR model can be interpreted as follows:


$$Y_{t}={\text{Π}}_{i,t}Y_{i,t-1}+\mu_{t};\mu_{t}\sim N\left(0,S_{t}\right)$$ (1)

$${\text{Π}}_{t}={\text{Π}}_{t-1}+\varepsilon_{t};\varepsilon_{t}\sim N\left(0,R_{t}\right)$$ (2)

Equ. (1) and Equ. (2) are the main part and qualification of the TVP-VAR model, respectively. Where $Y_{t}$ and $\mu_{t}$ are N×1 dimensional vector whereas the first one represents N market returns whereas the latter illustrates the error disturbance. ${\text{Π}}_{i,t}$ is an $N\times N_{p}$ coefficient matrix of time-varying VAR, and $Y_{i,t-1}$ is a $N_{p}\times 1$ vector which represents conditional dependent variables. The lower corner p represents the p-order lag. $S_{t}$ and $R_{t}$ are N×N and $N_{p}\times N_{p}$ time-varying variance-covariance matrices of the error vector $\mu_{t}$ and $\varepsilon_{t}$, respectively. In particular, $\varepsilon_{t}$ is an $N\times N_{p}$ dimensional error matrix.

Then, we need to transform the TVP-VAR into a TVP-VMA model, and the TVP-VMA model is as follows:


$$Y_{t}=\overset{\infty }{\underset{j=0}{\sum }}A_{jt}\mu_{t-j}$$ (3)

Equ. (3) is set up to obtain its coefficients and variance for the next step of generalised variance decomposition. where $A_{jt}$ is the N ×  N coefficient matrix.

Since the TVP-VMA model is unstructured, it is necessary to use the H-step-ahead forecast model to calculate the GFEVD. It is shown as follows:


$$\delta_{ijt}\left(H\right)=\frac{{\left(S_{t}\right)}_{jj}^{-1}\sum_{h=0}^{H}{\left({\left(A_{t}^{h}S_{t}\right)}_{ijt}\right)}^{2}}{\sum_{h=0}^{H}{\left(A_{t}^{h}S_{t}A_{t}^{h^{\prime }}\right)}_{ii}}$$ (4)

$${\tilde{\delta }}_{ijt}\left(H\right)=\frac{\delta_{ijt}\left(H\right)}{\sum_{k=1}^{N}\delta_{ijt}\left(H\right)}$$ (5)

Equ. (4) represents the impact of variable j on variable i after a shock. The numerator in Equ. (5) represents the impact of a shock to a single variable, while the denominator represents the combined impact from all variables. We set the forecast step size to 10, which is an appropriate length to help us capture both short-term and long-term trends. The rolling window we set to 200, which is equivalent to a full natural trading year, giving us a longer window to analyse data characteristics.

Further, ${\tilde{\delta }}_{ijt}\left(H\right)$ should be normalised so as to achieve a summation of each row of ${\tilde{\delta }}_{ijt}\left(H\right)$ equal to 1. Then, we can obtain the following equation: $\overset{N}{\underset{i=1}{\sum }}{\tilde{\delta }}_{ijt}\left(H\right)=1$ and $\overset{N}{\underset{j=1}{\sum }}\overset{N}{\underset{i=1}{\sum }}{\tilde{\delta }}_{ijt}\left(H\right)=N$.

Based on the above, we can then proceed to the calculation of the connectedness measure. We started with a measure of the net pairwise connectedness, which can be expressed as follows:


$$NPC_{ijt}\left(H\right)={\tilde{\delta }}_{ijt}\left(H\right)-{\tilde{\delta }}_{jit}\left(H\right)$$ (6)

If $NPC_{ijt}\left(H\right)>0$ in Equ. (6), it implies that variable j has a greater influence on variable i and vice versa.

We need to calculate the the total directional connectedness to others:


$$TO\left(H\right)=\overset{N}{\underset{i=1,i\neq j}{\sum }}{\tilde{\delta }}_{ijt}\left(H\right)$$ (7)

Equ. (7) represents the combined effect that variable j exerts on all other variables.

The total directional connectedness from the other variables should also be accounted for:


$$FROM\left(H\right)=\overset{N}{\underset{j=1,j\neq i}{\sum }}{\tilde{\delta }}_{jit}\left(H\right)$$ (8)

Equ. (8) represents the combined effect that variable i receives from all other variables exerted on it.

The net total connectedness index is expressed as follows:


$$NET\left(H\right)=TO\left(H\right)-FROM\left(H\right)$$ (9)

Equ.(9) represents the difference between the influence exerted by one variable on all other variables and the combined influence exerted on it by all other variables. If NET(H) >  0, it represents that the effect of variable j on the whole connectedness network is greater than the effect of the whole connectedness network on variable j, and vice versa.

The value of total connectedness can be calculated by the following equation:


$$TCI\left(H\right)=\frac{\sum_{j=1}^{N}TO\left(H\right)}{N}=\frac{\sum_{i=1}^{N}FROM\left(H\right)}{N}$$ (10)

Equ. (10) represents the mean value of the influence exerted by the variable on the connectedness network, or the mean value of the combined influence received by the variable from the connectedness network.

So far, we have mainly studied connectedness in the time domain, and next we will continue to study connectedness in the frequency domain. Based on Stiassny’s (1996) study of spectral decomposition methods, we can study connectedness in the frequency domain [70]. The equations are as follows:


$$A\left(e^{-iw}\right)=\overset{\infty }{\underset{h=0}{\sum }}e^{-iwh}A_{t}^{h}$$ (11)

Equ. (11) is a vector moving average model that incorporates frequency changes. Where $i=\sqrt{-1}$ and *w* is a specific frequency.


$$F_{y}\left(w\right)=\overset{\infty }{\underset{h=-\infty }{\sum }}E\left(Y_{t}Y_{t-h}^{'}\right)e^{-iwh}=A\left(e^{-iw}\right)S_{t}A^{\prime }\left(e^{+iw}\right)$$ (12)

Equ. (12) is the TVP-VMA model with Fourier transform performed.


$$\delta_{ijt}\left(w\right)=\frac{{\left(S_{t}\right)}_{jj}^{-1}|\sum_{h=0}^{\infty }{\left(A\left(e^{-iw}\right)S_{t}\right)}_{ijt}{|}^{2}}{\sum_{h=0}^{\infty }{\left(A\left(e^{-iw}\right)S_{t}A^{\prime }\left(e^{+iw}\right)\right)}_{ii}}$$ (13)

Equ. (13) is the GFEVD in the frequency band representing the combination of spectral density and generalised prediction error variance decomposition.


$${\tilde{\delta }}_{ijt}\left(w\right)=\frac{\delta_{ijt}\left(w\right)}{\sum_{k=1}^{N}\delta_{ijt}\left(w\right)}$$ (14)

Equ. (14) is a normalised treatment of the variance decomposition. Where ${\tilde{\delta }}_{ijt}\left(w\right)$ can be interpreted as the effect from jth variable to ith variable at a given frequency *w*.


$${\tilde{\delta }}_{ijt}\left(d\right)=\overset{w2}{\underset{w1}{\int }}{\tilde{\delta }}_{ijt}\left(w\right)dw$$ (15)

Equ. (15) represents the combined effect exerted by variable j on i given a specific frequency band d. We set the frequency bands into two parts, d = (π/5,π) for the high frequency band, corresponding to periods of 1-5 days, and d = (0,π/5) for the low frequency band, corresponding to periods of more than 5 days. A natural trading week of five days is appropriate as a boundary between short and long term.

Based on the above, we can obtain the equation about the connectedness index in a certain frequency band d as follows:


$$NPC\left(d\right)={\tilde{\delta }}_{ijt}\left(d\right)-{\tilde{\delta }}_{ijt}\left(d\right)$$ (16)

Equ. (16) represents the net pairwise connectedness for a given frequency band.


$$TO\left(d\right)=\overset{N}{\underset{i=1,i\neq j}{\sum }}{\tilde{\delta }}_{ijt}\left(d\right)$$ (17)

Equ. (17) represents the combined effect exerted by the j variable for a given frequency band on all other variables.


$$FROM\left(d\right)=\overset{N}{\underset{j=1,j\neq i}{\sum }}{\tilde{\delta }}_{ijt}\left(d\right)$$ (18)

Equ. (18) represents the influence received by variable i in a given frequency band from the influence exerted by all other variables.


$$NET\left(d\right)=TO\left(d\right)-FROM\left(d\right)$$ (19)

Equ. (19) represents the difference between the influence exerted on the connectedness network by a variable in a given frequency band and the influence exerted on it by the connectedness network.


$$TCI\left(d\right)=\frac{\sum_{j=1}^{N}TO\left(d\right)}{N}=\frac{\sum_{i=1}^{N}FROM\left(d\right)}{N}$$ (20)

Equ. (20) represents the mean value of the influence exerted by a variable on the connectedness network for a given frequency band, or the mean value of the combined influence received by the variable from the connectedness network.

### 3.2 Data

The main goal of this paper is to investigate the asymmetric spillover relationship between the clean power market and industrial markets classified by carbon emissions, and to explore the role of various uncertainties. Jiang et al. (2023) pointed out that in China’s clean energy composition, the most important are hydropower, wind power and solar power, which together account for more than 90% of clean energy [40]. Therefore, this article uses the stock indices of hydropower, wind power and solar power to represent the clean energy market. The hydropower (HY), wind power (WP) and solar power (SP) stock indices are represented by the CITIC Hydropower Index, the CITIC Wind Power Index and the CITIC Solar Power Index respectively. In order to reasonably obtain the portfolio of high-carbon, low-carbon and ordinary industrial markets, we refer to the practice of Zhang and Xu (2023), sorted 34 industries from low to high according to carbon emissions, and define those with carbon emissions less than 4 Mt as low-carbon portfolio (LC), those with more than 100 Mt as high-carbon portfolio (HC), and industries between 4 Mt and 100 Mt as ordinary portfolio (OR) [7]. The specific industries included in each portfolio are detailed in Appendix 1. For each included portfolio of industries, we used equal weighted averaging to calculate and obtain the available data. All data come from the Wind database and the data are processed by first order logarithmic difference, that is: $R_{t}=LN\left(P_{t}/P_{t-1}\right)$.

Based on data availability, the full period for the study is from 2 January 2014 to 9 October 2023. This period includes major events such as the Chinese stock market collapse that occurred in 2015, the U.S.-China trade conflict that occurred in 2018, the COVID-19 pandemic that occurred in 2022, and the Russian-Ukrainian conflict that occurred in 2022. Table 1 presents the descriptive statistical analysis and unit root tests for the regression series that are relevant to this study. On average, all variables have positive returns, with solar power having the highest return, followed by hydropower. A review of the standard deviations of each variable reveals that solar and wind power exhibit relatively high standard deviations, ranking first and second, respectively. The high-carbon portfolio, low-carbon portfolio, and ordinary portfolio follow in descending order, ranking third, fourth, and fifth, respectively. Overall, the standard deviations of the industrial stock markets are lower than those of the clean energy market, indicating that the returns of the industrial stock markets are more stable. All variables in the sample have negative skewness and kurtosis. The J-B statistic also confirms this result; all series are significant. Both the ADF and PP tests pass, indicating that the variables are smooth series and satisfy the conditions of the DY and BK methods.

**Table 1:** Descriptive statistics and unit root tests for each variable.

Variable	Mean	Std	Skewness	Kurtosis	J-B statistics	ADF test	PP test
HY	0.0005	0.0155	-0.752	13.97	12000***	-44.377***	-1798.71***
WP	0.0002	0.0226	-0.337	6.330	1142***	-46.669***	-1767.16***
SP	0.0007	0.0237	-0.358	5.941	906.3***	-45.451***	-1817.38***
LC	0.0003	0.0167	-1.085	10.32	5769***	-44.495***	-1708.87***
OR	0.0004	0.0164	-0.935	9.764	4871***	-44.879***	-1710.66***
HC	0.0004	0.0176	-0.973	9.334	4343***	-45.185***	-1751.84***

HY, WP, SP, LC, OR, and HC represent the hydropower market, wind power market, solar power market, low-carbon industrial market, ordinary industrial market, and high-carbon industrial market, respectively.

Table 2 provides a matrix of Pearson’s correlation coefficients, which shows that there is considerable correlation between HY, WP, SP, LC, OR, and HC. The highest correlation is between LC and HC, with a value of 0.936, and the lowest is between HY and WP, with a value of 0.483. The correlation between the industrial stock submarkets is markedly higher than that observed between the clean energy submarkets, and also higher than the correlation between the industrial stock submarkets and the clean energy submarkets.

**Table 2:** Pearson correlation matrix.

Variable	HY	WP	SP	LC	OR	HC
HY	1					
WP	0.533***	1				
SP	0.483***	0.745***	1			
LC	0.649***	0.781***	0.764***	1		
OR	0.638***	0.767***	0.778***	0.917***	1	
HC	0.676***	0.765***	0.759***	0.936***	0.877***	1

HY, WP, SP, LC, OR, and HC represent the hydropower market, wind power market, solar power market, low-carbon industrial market, ordinary industrial market, and high-carbon industrial market, respectively.

## 4. Empirical results

### 4.1 The spillover effects in the time domain and frequency domain

#### 4.1.1 Static spillovers in the time and frequency domain.

In order to investigate the spillover connectedness between clean power and industrial share markets, we employed the DY and BK approach to gain a table of connectedness between the markets, as showed in Table 3. The value of the TCI index for the network comprising the green energy market and the industrial stock market is 67.60%, which suggests a notable spillover effect between the variables within the network. This result is considerably higher than the empirical findings for crude oil and industrial stock markets, who determined that the total connectedness value was below 50% [7]. This is related to the choice of the Brent crude oil index as an representation of the crude oil market, which does not represent the trend of local crude oil prices in China. Furthermore, the occurrence of this result is also related to the fact that the data we have chosen for both the clean energy and the industrial sector are derived from the stock market. Huang and Liu (2023) demonstrated a greater degree of connectedness between variables within the same market [71]. Finally, it can be observed that the value of the short-term TCI (60.95 per cent) is significantly greater than the value of the long-term TCI (6.65 per cent). This implies that spillovers in the network are driven by the short run, with individual assets rapidly spilling over shocks in the short run.

**Table 3:** Static return connectedness.

	HY	WP	SP	LC	OR	HC	FROM
HY	52.42	7.39	6.51	10.45	10.55	12.67	47.58
	**47.58**	**6.6**	**5.88**	**9.44**	**9.5**	**11.42**	**42.85**
	***4.84***	***0.8***	***0.63***	***1.01***	***1.04***	***1.25***	***4.74***
WP	5.29	31.51	15.62	16.56	15.6	15.42	68.49
	**4.74**	**28.5**	**14.04**	**15**	**14.04**	**13.94**	**61.77**
	***0.55***	***3.02***	***1.58***	***1.55***	***1.56***	***1.48***	***6.72***
SP	4.71	15.6	31.04	16.22	16.84	15.58	68.96
	**4.22**	**14.11**	**28.02**	**14.73**	**15.12**	**14.16**	**62.34**
	***0.49***	***1.49***	***3.02***	***1.5***	***1.72***	***1.42***	***6.62***
LC	6.21	13.85	13.68	25.69	19.34	21.23	74.31
	**5.54**	**12.44**	**12.24**	**23.13**	**17.27**	**19.06**	**66.55**
	***0.67***	***1.41***	***1.44***	***2.56***	***2.07***	***2.18***	***7.77***
OR	6.31	13.6	14.78	20.21	27.36	17.73	72.64
	**5.71**	**12.31**	**13.4**	**18.36**	**24.68**	**16.06**	**65.83**
	***0.6***	***1.3***	***1.38***	***1.85***	***2.68***	***1.67***	***6.81***
HC	7.5	13.26	13.48	21.81	17.59	26.36	73.64
	**6.73**	**11.99**	**12.15**	**19.73**	**15.8**	**23.79**	**66.39**
	***0.77***	***1.27***	***1.34***	***2.07***	***1.8***	***2.57***	***7.25***
TO	30.02	63.71	64.08	85.25	79.92	82.64	
	**26.93**	**57.45**	**57.71**	**77.27**	**71.73**	**74.65**	
	***3.09***	***6.27***	***6.37***	***7.98***	***8.19***	***8***	TCI
Net	-17.57	-4.77	-4.88	10.94	7.28	9	67.60
	**-15.92**	**-4.32**	**-4.63**	**10.72**	**5.9**	**8.25**	**60.95**
	***-1.65***	***-0.45***	***-0.25***	***0.22***	***1.39***	***0.75***	***6.65***

HY, WP, SP, LC, OR, and HC represent the hydropower market, wind power market, solar power market, low-carbon industrial market, ordinary industrial market, and high-carbon industrial market, respectively. Normal font represents total spillover levels, bold represents short-term spillover levels, and italic represents long-term spillover levels.

The spillover values for the ‘TO’ rows indicate that spillovers in the industrial stocks submarkets categorised by carbon emissions are larger than spillovers in the clean energy submarkets. The low-carbon portfolio has the greatest spillover effect on the network, followed by the high-carbon portfolio and the ordinary portfolio, with values of 85.25%, 82.64% and 79.92%, respectively. In the clean energy submarkets, solar energy and wind energy have the fourth and fifth highest spillovers on the network, with 64.08% and 63.71%, respectively. In comparison, the spillover result for hydropower is much lower at 30.02%. A further examination of the shocks received by the variables from the network, also known as the ‘FROM’ column, reveals that the industrial stock market is also affected to a greater extent than the clean energy market. The low-carbon portfolio receives the most shocks from the network (74.31%), while hydro energy receives the least shocks from the network (47.58%). The low-carbon portfolio, which exerts the strongest influence on the connectedness network, also receives the strongest risk spillovers from the network. This represents a very strong link between the low carbon portfolio and other markets. In contrast, the water energy market that exerts the lowest shocks to the connectedness network also receives the lowest shocks from the network. It can be argued that hydro energy is more independent in the connectedness network. The potential reason for these two results is that hydro is a relatively mature and independent energy technology, whereas many sectors in the low-carbon portfolio need to interact and integrate with network technologies frequently and have more links to other markets. This implies that the low-carbon portfolio is hardly a hedging asset, whereas hydro energy is a good hedging asset.

We have divided the table into four sections, with the top left corner showing mutual spillovers between clean energy submarkets, the bottom right corner representing mutual spillovers between submarkets in the industrial stock market, the top right corner showing spillovers from the industrial stock market to the clean energy market, and the bottom left corner representing spillovers from the clean energy market to the industrial stock market. Comparing the top left corner to the bottom right corner, it is clear that the bottom right corner has the better value. This represents that the connectedness between the submarkets of the industrial stock market is stronger than the connectedness between the clean energy submarkets. The low carbon portfolio, the high carbon portfolio and the ordinary portfolio are more strongly connected in the stock market than hydro, wind and solar. A comparison of the top right and bottom left corners of the table shows that the influence exerted by the industrial stock market on the clean energy market is stronger than the influence exerted by the clean energy market on the industrial stock market. This represents the fact that the industrial stock market dominates the entire network. This is in line with our expectations, as China’s industrial stock market not only has more participants than the clean energy market, but also has deeper linkages between internal sectors. Clean energy is also relatively independent in terms of stock market performance due to its relatively mature technology. As a result, the industrial stock market can be used as a risk indicator, and when the industrial stock market is in turmoil, a financial storm sweeping through the industrial and clean energy sectors will follow.

As shown in the ‘NET’ row of Table 3, the submarkets in the industrial equity market have positive net spillovers, while the submarkets in the clean power market have net pass-through effects of less than zero. The Low Carbon Portfolio, High Carbon Portfolio, and General Portfolio are net senders of risk premiums, while Hydro, Wind, and Solar are net receivers of risk premiums. The highest net spillover is in the low carbon portfolio (10.94%) and the lowest net spillover is in hydro (-17.57%). This is an interesting finding, with the low carbon portfolio being the largest net sender to the connectedness network and hydro being the largest net receiver. The turbulence that occurs in the low carbon portfolio will have the greatest impact on the other markets, while hydro energy passively absorbs the impact from the other markets. Zhang and Xu (2023) argue that China’s stock market is policy-driven, and as low-carbon policies continue to be implemented, low-carbon industries are receiving more attention and are more likely to generate returns for investors, making low-carbon portfolios the main sender of connectedness networks [7]. Investor attention also means that low carbon portfolios have more volume; other sectors may struggle to drive movement in low carbon portfolios, but changes in the low carbon sector will have a significant impact on other sectors. Although hydro energy is the most important clean energy source in China, its technology is relatively mature, making it difficult to enjoy changes in share prices due to technological change, and its market share is relatively stable. These characteristics determine that the main investors in hydro energy are long-term investors who do not easily sell their shares and invest in other markets. But relatively, when there are abnormal changes in other markets, some investors will flock into or out of the water energy market, causing turbulence in the water energy market.

Considering the frequency bands of connectedness, the industrial stock market is the net sender and the clean market is the receiver in both the high (short-term) and low (long-term) frequency bands. In Table 3, The normal font is for the full band spillover values, and the fonts for the high and low band values are bold and italic, respectively. In the industrial stock market, the low-carbon portfolio is the primary net sender of risk in the high frequency band and the secondary net sender of risk in the low frequency band. This suggests that the low carbon portfolio exerts a major impact on the network from the short term, and in the long term, the low carbon portfolio exerts a lower impact on the network compared to the high carbon portfolio as well as the normal portfolio. Low-carbon portfolio, as the most powerful emitter of risk, will always be able to quickly change the structure of the rest of the market in a short period of time, completing the transfer of risk. The high carbon portfolio as well as the portfolio are not only weaker than the low carbon portfolio, but are also impacted by the low carbon portfolio, so the risk spillover is more in the long term. The most visible of the clean energy markets is still the hydro energy market, which is the strongest risk receiver in both the short and long term. The hydro energy market is the strongest risk-receiving pool, and the risk of spillovers from all other markets is largely absorbed by the hydro energy market, so it is subject to major shocks from other markets in both the short and long term. In addition, it is a key finding that the impact of any market on the network and the shocks received from the network are mainly from the short term. This means that in the network, information is processed quickly and then quickly spills out of each variable and into all others. According to the fully efficient market hypothesis, changes in share prices reflect all valid information, but China’s stock market takes five days to process the information to a greater extent, which in challenges the efficient market hypothesis and supports the behavioural finance explanation of the market.

#### 4.1.2 Dynamic total spillovers in the time and frequency domain.

**Fig 1:**
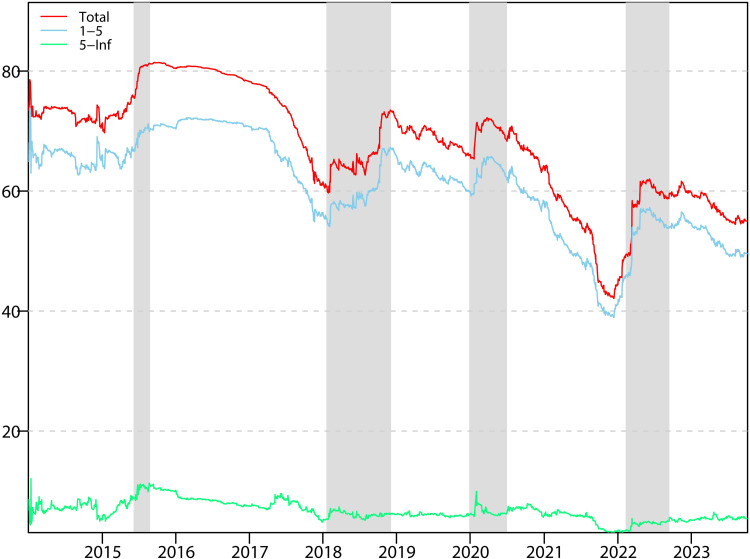
Dynamic total spillover.

We have analysed the static connectedness between the clean power and the industrial stock market above, but since the static connectedness is only an average over the sample period, it does not reflect the relationship between the markets over time. Experience has shown that spillovers between markets change as a result of changes in the economic environment, the political environment and even the public health environment, so we believe that a discussion of dynamic connectedness is warranted. In order to measure the dynamic spillovers between our chosen variables and to further uncover the relationships between the variables, we use a rolling window and split frequency bands. The rolling window is 200 days and the forecasting step is 10 days. The frequency bands are divided into high and low frequencies, corresponding to short term (1-5 days) and long term (more than 5 days). The results are presented in Fig 1.

The spillover index rises rapidly after the Chinese stock market crash, peaking at 82% throughout the sample period. And the impact of the stock market crash lasted for a long time until early 2017, when the spillover index started to decline at an accelerated rate. This suggests that the Chinese share market crash has resulted in a notable intensification of the connectedness between the clean energy market and the industrial stock market. As might be expected, the variables selected are associated with the Chinese stock market. It is therefore unsurprising that a rise in the spillover index is accompanied by a corresponding increase in the volatility of the share market. A stock market crash represents a significant setback for investor confidence, which is likely to result in a prolonged period of caution among investors with regard to further investment in the share market.

It can be observed that the connectedness index demonstrates two distinct periods of accelerated growth during the period of the US-China trade conflict. The first of these occurred in January and early February 2018, following the announcement by the US of global safeguard tariffs on imported solar panels. The China Photovoltaic Industry Association (2018) released a report indicating that China accounts for 67 per cent of the total global cell production and 71 per cent of the total global module production. Consequently, the imposition of tariffs on solar panels by the US will have a detrimental impact on the interests of China’s PV-related industries. In response, China implemented tariffs on a range of US goods, initiating a trade conflict between the two major economies. This trade conflict has resulted in an increase in the spillover index to 65 per cent. The second phase of the trade conflict commenced in September 2018, when the United States announced tariffs on approximately $200 billion of Chinese imports, and China responded by increasing tariffs on approximately $60 billion of US goods. This intensified the impact of the trade conflict, resulting in a peak in the connectedness index for the period (approximately 72%).

In the wake of the global outbreak of the novel coronavirus, a substantial corpus of literature has appeared, underscoring the profound economic impact of the pandemic [72,73]. It is evident that the outbreak led to a surge in the spillover index between markets [74,75], a finding that aligns with our own observations. In the early months of 2020, when the outbreak first emerged, the total spillover index exhibited a notable acceleration, rising from 65% to 70% in a relatively short period. This implies that the outbreak of the COVID-19 had a profound influence on the clean energy and industrial stock markets, with a concomitant increase in market risk. Subsequently, the connectedness index declined as the outbreak was effectively contained and the Chinese economy rebounded rapidly. In the aftermath of the global pandemic, the global economy experienced a decline, with financial markets in all countries experiencing turbulence. The risk spillover index increased, and market risk premiums intensified. This is evidenced by the spillover correlation between clean energy market and the industrial stock market.

At the end of 2021, the spillover index begins to rise after a period of minimal activity (42%). From this point onwards, clashes begin to emerge on the Russian-Ukrainian border, and war becomes increasingly likely. In particular, following the outbreak of the Russia-Ukraine conflict, the spillover index reached a new peak of 58%. It is evident that the Russian-Ukrainian conflict has an influence on the spillovers between the selected markets, with an increase of 10 per cent, which is the highest among the four major emergencies. It is evident that the occurrence of large wars in neighbouring countries has the most significant impact on the spillovers between markets. As a result, market participants must be concerned about possible wars in China’s neighbouring countries, which could have a huge impact on the market.

The four key periods we study represent the impact of financial crises, real economic crises, trade crises and geopolitical crises on markets. Among the four key periods, the level of total connectedness is highest during the Chinese stock market crash, followed by the pandemic period, and then by the US-China trade conflict and the Russia-Ukraine conflict. This implies that this financial and economic crisis had the greatest impact on the markets we study. The market we study is actually part of the stock market, which is an important part of the financial market, so the financial crisis will have the most direct impact on the stock market, as evidenced by the extremely high level of connectedness of the market network. Financial markets serve the real economy, and the outbreak of COVID-19 greatly disrupted China’s real economy, and the economic turmoil was transmitted to the financial sector, resulting in turmoil in the financial markets and a rise in the connectedness between stock markets. The U.S.-China trade conflict has had a significant impact on China’s commodity exports, and the decline in exports has affected China’s real economy, leading to an increase in connectedness. Moreover, the trade conflict between the U.S. and China has a tendency to intensify, which could easily lead to investors’ disillusionment with the Chinese market, and the lowering of expectations can also affect the financial market. The Russian-Ukrainian conflict has the lowest level of connectedness because there is not much direct connection between the Russian-Ukrainian conflict and China. The main reason for the increase in the level of market connectedness due to the Russian-Ukrainian conflict may be attributed to investors’ fear of World War III.

Examination of the frequency bands shows that the level of total spillover connectedness is consistently and significantly higher in the high-frequency bands (blue line) than in the low-frequency bands (green line). The proportion of high-frequency connectedness fluctuates between 40% and 70% over the entire sample period, while the proportion of low-frequency spillovers fluctuates between 5% and 10%. The high volatility of high-frequency connectedness is suggestive of the vulnerability of the Chinese stock market and the risk of causing short-term turbulence in the Chinese stock market on a regular basis. In addition, the study also finds that short-term aggregate spillover connectedness is highly correlated with the aggregate spillover index, especially in the case of sudden large-scale events. This proves that short-term aggregate spillovers dominate the entire market, especially in the case of unexpected major events, where risks spill over quickly and cause unimaginably heavy damage to the entire network. In contrast, the long-term connectedness index shows greater stability. This suggests that major events and minor surprises mainly affect short-term market trajectories, with minimal impact on the long term. In terms of frequency band performance over the four key periods, the highest total short-term spillover effect is observed during the Chinese crash, representing a period of great risk, cumbersome information, and constant shock to the stock market. Moreover, the performance of the Chinese stock market crash in terms of connectedness in the low frequency bands is also noteworthy, with spillover effects also being the highest in the four periods. The occurrence of a stock market crash not only causes market turmoil in the short term, but its effects continue to be felt for a long period of time before the market fully digests them. The Russian-Ukrainian conflict had the lowest impact on the market in both the short and long term. This is because the Russian-Ukrainian conflict took place on the border between Russia and Ukraine, and China is not connected, the fire did not spread to the Chinese border. As for why the Russia-Ukraine conflict could affect China’s stock market, we suspect it’s because people are worried that the Russia-Ukraine conflict could be a trigger for World War III. And the impact of war on financial markets is extremely scary [21].

#### 4.1.3 Dynamic net spillovers in the time and frequency domain.

**Fig 2:**
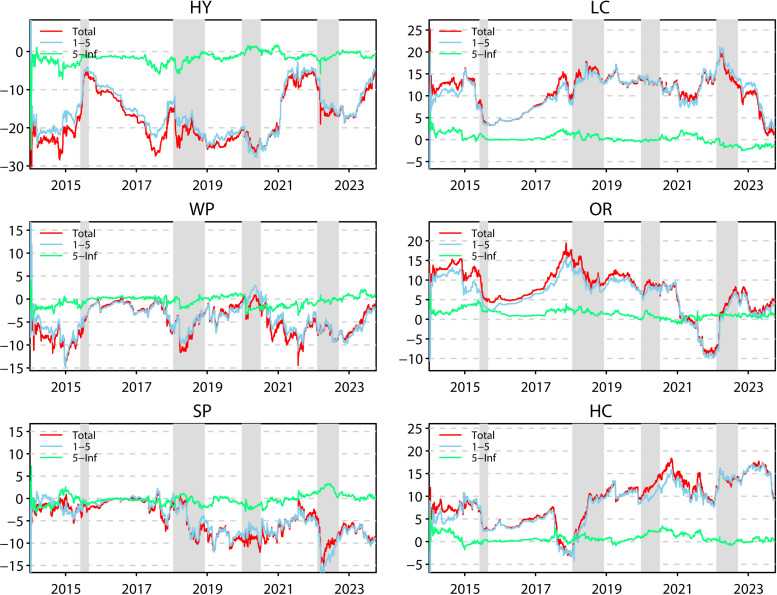
Net return spillover.

The dynamic total spillover connectedness index represents the overall average of the network of selected variables. However, this index does not permit the observation of the role played by each submarket separately. Consequently, it is necessary to analyse the net connectedness index. The role played by each submarket will also be examined in both the time and frequency domains, in a manner analogous to the analysis of the dynamic total spillover index. Fig 2 presents the dynamic net spillover index for each market, offering a comprehensive visual representation of the direction of spillovers in each market.

The clean energy submarket is a net recipient throughout the sample period, and even if occasionally the spillover value is greater than zero, it immediately turns around and returns to a value less than zero. This is consistent with our conclusion from analysing static spillovers that the clean energy market is a recipient of risky spillovers in the network. In addition, our results show that hydropower is the most volatile, with fluctuations of up to -30% and zero. This highlights the high sensitivity and volatility of the hydropower market to changes in the overall network. Of the four key periods, hydro’s performance during the crash is noteworthy. Hydro Energy’s net risk acceptance declined in the wake of the crash. The onset of a financial crisis can trigger panic among investors. While at other times hydroenergy has less of an impact on other markets, as panic spreads, hydroenergy in turn increases its impact on other markets. This then leads to a decrease in the net risk acceptance of the water energy market. In the other three periods, the hydro energy market played its full role as a risk absorber as it was not directly linked to the crises represented in these three periods. Wind energy’s performance during the U.S.-China trade conflict is the most noteworthy, with its initial performance as a strong net risk taker followed by a rapid decline in capacity. Both China and the US are leaders in wind energy, and the trade conflict between the US and China has partly centred around wind products, which has directly impacted the Chinese wind market, causing it to also increase its impact on the network, which in turn has resulted in a decline in the net risk-receiving capacity of wind energy. Similarly to hydro, the wind market’s net risk-absorbing capacity weakened during the stock market crash, acting as a risk receiver during the pandemic and during the Russian-Ukrainian conflict. Solar’s performance during the four pandemics is also noteworthy, as it demonstrated its strong risk-absorbing capacity during both the U.S.-China trade conflict and the Russian-Ukrainian conflict, both of which saw a sudden increase in risk absorption followed by a decline. This is related to solar’s role as a risk receiver, where clean energy is forced to absorb risk from the market network when it is hit. And as events unfold and the market stabilises, the risk absorbed by clean energy declines, as does the risk absorbed by solar. The Russian-Ukrainian conflict strained the raw materials associated with the solar industry, and the solar market suddenly increased its risk spillover to the network, causing a sudden decline in its role as a risk-absorbing pool.

In the industrial stock market, the ordinary and high-carbon portfolios act as net senders of risk for the vast majority of the period, and the low-carbon portfolio is a net sender of risk for the entire sample period. Consistent with Zhang and Xu’s study (2023) [7], as the Chinese government increases its support for low-carbon emitting industries, high-carbon portfolios continue to be subject to risk spillovers from both low-carbon and ordinary portfolios, forcing them to become net receivers of network. Whereas the shift to high-carbon sectors takes a significant amount of time, as the emotional impact of the policy wanes, high-carbon portfolios gradually increase their influence and turn back into net risk spillovers. The ordinary portfolio begins to turn into a net recipient of risk in 2021, a time period in which the COVID-19 pandemic has been effectively contained, the Chinese economy is experiencing a rebound, and the ordinary portfolio rapidly reduces its risk spillover to the network while continuing to receive risk spillover from other markets. At the onset of the crisis, industrial stock market performance was similar across carbon emissions. All three portfolios experienced a decline in net spillover capacity during the crash, due in large part to the diminished ability of clean energy to act as a risk absorber and to spill over greater risk to industrial equity markets. In the other three important periods, all three portfolios acted as strong risk emitters. As investor sentiment stabilised, the industrial equity submarkets exerted less influence on the network and the net spillover effect diminished.

An analysis of the performance of the spillover indices across the frequency bands indicates that the long-term net spillover index remains at approximately zero in all six sub-markets, while the short-run net spillover connectedness index demonstrates considerable fluctuation, essentially aligning with the performance of the total net spillover connectedness index. This suggests that short-term spillovers play a dominant role in each market, consistent with the previous analysis. Similarly, we can observe the changes in the short-term net spillover index and the long-term net spillover index over the four crisis periods. It is evident that the short-term net connectedness indices of the six markets behave broadly in line with the total net spillover indices over the four periods, i.e., when a crisis occurs, markets react quickly to spillover risk to the network and to receive spillover risk from other markets. The behaviour of each market’s long-term spillover index in times of crisis is more complex. Some have stable long-trem spillover indices while short-term and total net spillover indices spike, such as the performance of industrial stock submarkets during the Russia-Ukraine conflict; some have long-term spillover indices that move in the opposite direction of total net spillover indices, such as the hydropower during the COVID-19 pandemic; and some have long-run connectedness indices that behave in line with long-term and total net spillover indices, such as clean power markets during the trade conflict. clean energy submarket during the trade conflict. Furthermore, it can be observed that industrial stock submarkets experience a significant increase in short-term net spillovers in times of crisis, while long-term net spillovers are stable, implying that industrial stock submarkets rapidly spill risk into the network in the short term, with spillovers outweighing the risk received from the network. The long-run net spillover connectedness index of the clean power submarkets basically moves together with the short-term net spillover index, implying that the clean energy submarkets are more permanently affected by the crisis shocks and have more uncertainty about the future than the industrial stock submarkets.

### 4.2 Asymmetric spillover effects in the time and frequency domain

#### 4.2.1 Asymmetric static spillovers in the time and frequency domain.

In order to study the asymmetric spillover effects of the network, we further obtain the spillover tables for different frequency bands, both for positive and negative series. Table 4 shows the asymmetric spillovers between markets for the full frequency band, while Tables 5 and 6 show the asymmetric spillover matrices for networks in the high frequency band (short term) and low frequency band (long term), respectively. Values without parentheses are positive spillover and those with parentheses are negative spillover. Table 4 shows that the overall bad spillovers are higher than the overall good spillovers for the full frequency bands, their values are 68.84% and 61.87% respectively. This result shows that the overall connectedness of the network is asymmetric and that the bad news is a bigger shock to the network. Notably, the spillovers that each market receives (transmits) from the network are heterogeneous and exhibit asymmetry under good news shocks and bad news shocks. Spillovers from all six markets to the network exhibit stronger bad than good returns, while spillovers from the network to each market, except for LC, also exhibit stronger bad than good spillovers. That is, all markets except the low-carbon portfolio are more sensitive to bad news, which is in line with the research by Zhang and Xu (2023) [7]. The result that markets are more drastically affected by bad news supports the idea of loss aversion in behavioural finance. Investors are more averse to losing gains of equal value than they are to gaining gains of equal value. For the low-carbon portfolio, good news receives more spillovers from the whole network compared to bad news, which is an interesting finding. In terms of net spillovers, the industrial stock submarkets are net transmitters and the clean energy submarkets are net receivers, both under positive and negative shocks. That is, the industrial stock submarkets transmit more risk to the network, regardless of whether it is good message or bad message, while the clean energy submarkets act as risk absorbers for the network.

**Table 4:** Asymmetric static connectedness in the full frequency band.

	HY	WP	SP	LC	OR	HC	FROM
HY	60.63	6.02	5.48	8.8	8.65	10.41	39.37
	(46.86)	(8.48)	(8.19)	(11.14)	(11.9)	(13.42)	(53.14)
WP	4.76	38.21	15.58	14.42	14.18	12.85	61.79
	(6.23)	(30.38)	(15.24)	(16.9)	(15.54)	(15.72)	(69.62)
SP	4.27	14.95	37.52	14.59	14.95	13.72	62.48
	(6.17)	(15.45)	(31.23)	(15.68)	(16.55)	(14.91)	(68.77)
LC	5.26	11.33	11.8	29.66	20.45	21.5	70.34
	(4.28)	(9.74)	(9.96)	(25.84)	(17.59)	(18.72)	(60.29)
OR	5.19	11.56	12.57	21.48	31.7	17.51	68.3
	(7.62)	(13.91)	(14.68)	(19.29)	(27.2)	(17.3)	(72.8)
HC	6.41	10.69	11.63	22.56	17.68	31.03	68.97
	(8.56)	(13.83)	(13.14)	(21.27)	(17.24)	(25.95)	(74.05)
TO	25.89	54.56	57.06	81.85	75.91	75.98	
	(35.74)	(66.22)	(64.8)	(84.28)	(79.87)	(82.15)	TCI
Net	-13.47	-7.23	-5.42	11.51	7.6	7.01	61.87
	(-17.4)	(-3.4)	(-3.96)	(9.59)	(7.06)	(8.11)	(68.84)

HY, WP, SP, LC, OR, and HC represent the hydropower market, wind power market, solar power market, low-carbon industrial market, ordinary industrial market, and high-carbon industrial market, respectively. Values inside parentheses are negative spillovers, values without parentheses represent positive spillovers.

**Table 5:** Asymmetric static connectedness in the high frequency band.

	HY	WP	SP	LC	OR	HC	FROM
HY	53.27	4.85	4.21	7.17	7.04	8.73	32.01
	(41.52)	(7.24)	(7.01)	(9.69)	(10.33)	(11.75)	(46.02)
WP	3.85	33.57	13.14	12.3	12.07	11.14	52.51
	(5.29)	(27.1)	(13.35)	(14.99)	(13.64)	(13.9)	(61.16)
SP	3.31	12.83	32.64	12.3	12.55	11.79	52.77
	(5.16)	(13.55)	(27.64)	(13.89)	(14.52)	(13.21)	(60.33)
LC	4.28	9.74	9.96	25.84	17.59	18.72	60.29
	(6.12)	(12.75)	(11.86)	(22.43)	(16.33)	(18.37)	(65.43)
OR	4.29	9.99	10.76	18.69	27.82	15.25	58.98
	(6.57)	(12.25)	(12.97)	(17.17)	(24.12)	(15.35)	(64.3)
HC	5.35	9.33	10.02	19.71	15.31	27.26	59.71
	(7.4)	(12.18)	(11.54)	(18.93)	(15.17)	(23.09)	(65.22)
TO	21.07	46.74	48.1	70.17	64.57	65.64	
	(30.54)	(57.96)	(56.73)	(74.67)	(69.99)	(72.57)	TCI
Net	-10.95	-5.77	-4.68	9.88	5.59	5.92	52.71
	(-15.48)	(-3.2)	(-3.6)	(9.24)	(5.69)	(7.36)	(60.41)

HY, WP, SP, LC, OR, and HC represent the hydropower market, wind power market, solar power market, low-carbon industrial market, ordinary industrial market, and high-carbon industrial market, respectively.Values inside parentheses are negative spillovers, values without parentheses represent positive spillovers.

**Table 6:** Asymmetric static connectedness in the low frequency band.

	HY	WP	SP	LC	OR	HC	FROM
HY	7.37	1.17	1.27	1.63	1.61	1.68	7.35
	(5.34)	(1.24)	(1.18)	(1.45)	(1.57)	(1.68)	(7.12)
WP	0.91	4.64	2.44	2.12	2.11	1.7	9.28
	(0.93)	(3.28)	(1.89)	(1.91)	(1.9)	(1.82)	(8.46)
SP	0.97	2.12	4.87	2.3	2.4	1.93	9.71
	(1.01)	(1.9)	(3.6)	(1.79)	(2.03)	(1.7)	(8.44)
LC	0.98	1.6	1.83	3.82	2.86	2.78	10.05
	(1.03)	(1.8)	(1.69)	(2.87)	(2.31)	(2.43)	(9.26)
OR	0.91	1.56	1.81	2.79	3.87	2.26	9.32
	(1.06)	(1.66)	(1.71)	(2.12)	(3.07)	(1.95)	(8.5)
HC	1.06	1.37	1.62	2.85	2.36	3.77	9.26
	(1.16)	(1.65)	(1.61)	(2.34)	(2.06)	(2.86)	(8.83)
TO	4.82	7.82	8.96	11.68	11.34	10.34	TCI
	(5.2)	(8.26)	(8.07)	(9.61)	(9.88)	(9.58)	
Net	-2.53	-1.46	-0.75	1.64	2.01	1.09	9.16
N	(-1.92)	(-0.2)	(-0.36)	(0.35)	(1.38)	(0.75)	(8.43)

HY, WP, SP, LC, OR, and HC represent the hydropower market, wind power market, solar power market, low-carbon industrial market, ordinary industrial market, and high-carbon industrial market, respectively. Values inside parentheses are negative spillovers, values without parentheses represent positive spillovers.

Similarly, the spillover effects in different frequency bands are asymmetric. Table 5 shows that the total spillovers in the higher frequency bands are 52.71 per cent and 60.41 per cent for positive and negative shocks, respectively. This indicates that spillovers are more susceptible to unfavourable information in the short run. Bad news brings panic to investors, which quickly spreads across markets and causes market turmoil. Table 6 shows that the total spillovers from positive and negative shocks in the low frequency band are 9.16% and 8.43%, respectively. In the long run, good news can generate sustained positive sentiment in the market. The persistence of this positive sentiment is stronger than the negative sentiment from bad news. It is worth noting that this difference between the high and low frequency bands is also reflected in the TO and FROM columns. In the high frequency band, the impact of bad news has a greater impact, both in terms of transmitting risk to the network and receiving risk from the network. In the low frequency band, on the other hand, good news dominates. In terms of net spillovers, the industrial equities submarket is a net sender and the clean energy submarket is a receiver of risk, regardless of which frequency band it falls into.

#### 4.2.2 Asymmetric total spillovers in the time and frequency domain.

**Fig 3:**
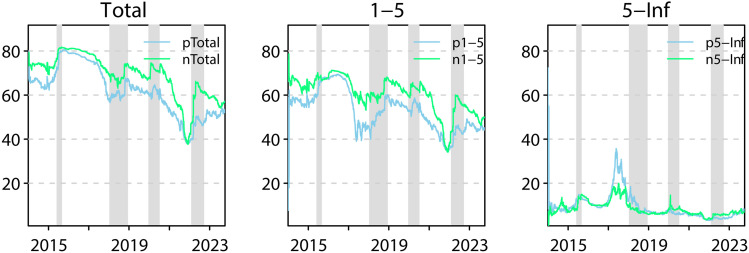
Asymmetric dynamic total spillover in different frequency bands.

Fig 3 provides a comparison of the influence of positive and negative shocks on total connectedness over time at different frequencies. It is clear that both the full frequency band and the short-term aggregate spillovers are dominated by negative returns over the sample period. Spillovers in the long-term are different, with spillovers from positive returns being stronger than spillovers from negative returns. Regardless of the frequency band they belong to, the spillover indices show asymmetry over time. The same can be observed for positive and negative returns in times of crisis, and it can be observed that the full frequency band and the short-run spillovers are similar in that there is a sudden rise in times of crisis. The long-run spillovers, on the other hand, are relatively moderate in the crisis period compared to the short-run spillovers. That is, the shocks from the crisis were absorbed in the short term and had little impact in the long term, a finding consistent with the previous section. Moreover, in terms of long-term spillovers, it can be found that good news brings more long-term shocks than bad news, because the spillover connectedness of positive returns is larger than that of negative returns.

Good news and bad news perform essentially similarly in the four key periods across frequency bands. Consider first the performance of the four periods in terms of total spillovers. During a stock market crash, good news and bad news cause about the same shocks, with the bad news perhaps a little ahead. In this period, the financial catastrophe tied all financial markets together so that good news and bad news had almost the same impact on markets. In the other three periods, shocks caused by bad news are in the lead, due to the fact that markets react more violently to bad news. The spillovers in the high and low frequency periods behave in the same way as the total spillovers, with bad news leading the way, but the difference is that the difference between the spillovers from shocks to the market caused by bad and good news is greater in the high frequency period than in the low frequency period. This is mainly due to the fact that spillovers from good and bad news are dominated by high-frequency spillovers.

#### 4.2.3 Asymmetric net spillovers in the time and frequency domain.

**Fig 4:**
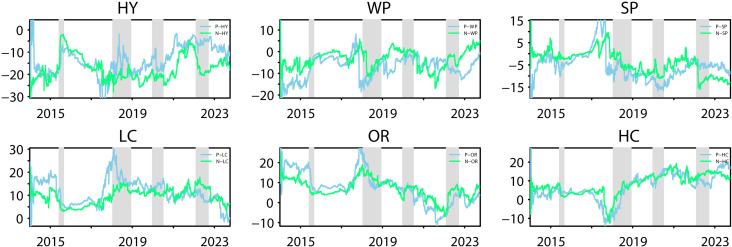
Asymmetric net return spillovers in the full frequency band.

**Fig 5:**
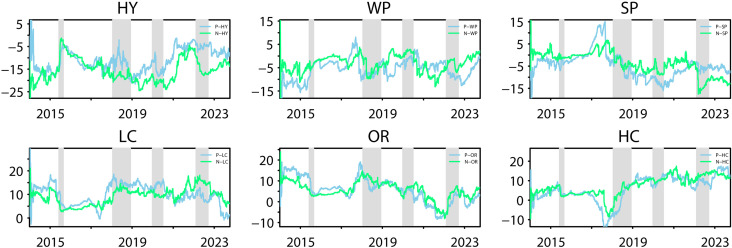
Asymmetric dynamic return spillover in high frequency bands.

**Fig 6:**
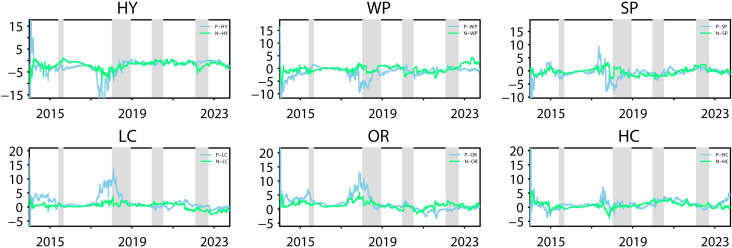
Asymmetric dynamic return spillover in low frequency bands.

The net spillovers over time for different markets under positive and negative shocks are shown in Fig 4, while Figs 5 and 6 represent the positive and negative net connectedness for each variable under different frequency bands. Fig 5 shows the findings in the high frequency band and Fig 6 shows the findings in the low frequency band. It is worthwhile to identify the dominant spillover effect in each market over time, so that we can find out which market is most affected by spillovers in response to good news (bad news) shocks. The larger the absolute value of the connectedness index, the greater the impact on the market of the positive (negative) shock represented by that spillover index. Among the green energy submarkets, it is clear that the hydropower is dominated by negative spillovers for long periods of time, and that the hydro market is a receiver of risk from the system network. This suggests that when bad news hits the hydro market, the hydro market will be hit harder by the network. The wind and solar markets, on the other hand, are dominated by positive spillovers. That is to say that good news shocks the wind and solar markets, causing these two markets to absorb more risk from the network. For the industrial stock submarkets, the low-carbon portfolio is found to be overwhelmingly dominated by positive spillovers most of the time, while it is difficult to tell who is in the lead for the ordinary and high-carbon portfolios. Therefore, when there is good news for the low-carbon portfolio, more attention needs to be paid to the impact it has on the systemic network.

Examination of the net positive and negative spillover indices for each market over different frequency bands shows that the short-term indices trend in line with the full-frequency indices. Hydropower markets are dominated by negative spillovers, while wind and solar markets and low-carbon portfolios are driven by positive spillovers in most cases. In the lower frequency band, i.e., the long-term spillovers, we note a moderate trend in the green line (bad news) and a significantly larger magnitude in the blue line (good news). This suggests that spillovers between the clean power submarket and the industrial equities submarket, driven by good news, are more noteworthy in the long run. In addition, the behaviour of positive and negative spillovers over four key periods in different frequency bands is also of interest. Hydropower is more risk-absorbent in good news shocks during the crash, but the effect is less pronounced. In the other three periods, it shows a clear dominance of negative spillovers. Water energy is more vulnerable to bad news shocks during crisis periods, with the exception of the financial crisis. The financial crisis made the financial markets so interconnected that both good and bad news hit the hydro market very hard. Wind is dominated by positive spillovers in all four periods, which is an interesting finding, as good news strengthens the risk-absorbing capacity of wind. Solar is more risk-absorbent in the presence of good news in the first three periods, while risk-absorption is stronger in the presence of bad news during the Russia-Ukraine conflict. It may be that bad news during the Russia-Ukraine conflict had a greater impact on other markets, leading to enhanced risk spillovers from other markets to the solar market. Similarly, the low-carbon market is dominated by good news shocks in the first three periods, acting as a risk transmitter and exerting influence on other markets. During the Russian-Ukrainian conflict, it was dominated by shocks caused by bad news, which acted as an emitter of bad news causing enhanced risk spillovers from the low carbon market to other markets. The performance of the high and average carbon markets is indistinguishable across the four periods, with both showing an equalisation of the spillover effects of good and bad news on market shocks. The high frequency period is the same as the total spillover effect, while the low frequency period is an equilibrium of net spillovers due to good news and bad news during the stock market crash, during the pandemic, and during the Russian-Ukrainian conflict, with risk spillovers due to good news stronger than to bad news during the U.S.-China trade conflict. The potential reason for this may be that the arrival of good news can enhance the risk spillover from clean energy to the industrial stock market.

### 4.3 The roles of uncertainties in spillover effects

#### 4.3.1 Selection of variables in the regression equation.

To ascertain the impact of uncertainties on spillovers between these markets, this paper incorporates uncertainties and total spillovers into the regression equation for an OLS regression. We select three variables, global economic policy uncertainty (EPU), global geopolitical uncertainty (GPR), and climate policy uncertainty (CPU), as explanatory variables, the Arca Tech 100 index (PSE), the CSI 300 index (CSI), the US dollar exchange rate against the renminbi (FX), and a dummy variable (DR) as control variables, and the different frequency bands (full frequency, high frequency, and low frequency) of total connectedness as dependent variables for the regression of the equation. The dummy variables are those assigned a value of 1 for time of crisis and 0 for non-crisis periods. Of course, the processing of the raw data is necessary, and we take the monthly means of all the data and treat them logarithmically except for the dummy variables, which are unchanged. Considering the availability of data, we select the monthly data from January 2014 to August 2023 for the study. The EPU index was developed by Baker et al. (2016) and there is evidence that it has an impact on financial markets and that economic policy uncertainty has a positive effect on connctedness [76]. The GPR index is derived from Caldara and Iacoviello (2022) and is based on their count of newspaper articles on geopolitical tensions [77]. Feng et al. (2023) showed that geopolitical risk triggers an upward trend in the level of market spillovers, due to the fact that risky events can exacerbate market price volatility [78]. Finally, the CPU index constructed by Gavriilidis (2021) affects price volatility in financial markets by influencing investors’ expectations [79]. The choice of control variables refers to the studies in these articles [6,18].

#### 4.3.2 How uncertainties affect the spillover effect.

Table 7 shows the regression results for uncertainty on aggregate and asymmetric spillovers, and it is notable that not all uncertainty has a significant effect on spillovers, and that different aggregate spillovers behave differently. A review of the regression results for total spillovers reveals that, with the exception of GPR, the remaining variables exert a notable influence on total spillovers. Among the factors under consideration, EPU has been identified as exerting a considerable positive influence on the total spillover effect. This implies that an increase in EPU is associated with a corresponding rise in the interconnectivity between the green energy market and the industrial share market. The finding of this result supports the idea of the herd effect in behavioural finance, where investors may tend to mimic the trading behaviour of others in order to reduce their own decision-making pressure when market uncertainty increases, and this herd effect leads to a rise in linkages between different sectors. CPU also significantly affects aggregate spillovers, but in the opposite direction. That is, concerns about climate issues can lead to a strengthening of the market’s spillover to itself. This is an interesting finding, as some of the literature indicates that CPU causes spillovers between markets to rise [54,79]. Among the control variables, PSE affects aggregate spillovers in the reverse direction, while the other variables CSI, FX and DR all have a positive contribution to aggregate spillovers. All the significant variables hold at 1% level of significance except DR, where the original hypothesis is rejected at 10% confidence interval.

**Table 7:** Regression results of uncertainties on spillover.

	Total	Positive	Negative
EPU	0.111***	0.0224	0.0992***
	(4.52)	(0.98)	(3.12)
GPR	-0.0260	-0.0275	-0.0401
	(-1.40)	(-1.32)	(-1.62)
CPU	-0.0463***	-0.0371**	-0.0529**
	(-2.94)	(-2.47)	(-2.33)
PSE	-0.461***	-0.590***	-0.480***
	(-24.64)	(-27.65)	(-19.04)
CSI	0.120***	0.233***	0.159***
	(4.97)	(7.36)	(5.38)
FX	0.848***	1.522***	1.029***
	(4.71)	(9.15)	(3.99)
DR	0.0167 *	0.00383	0.0351**
	(1.69)	(0.35)	(2.47)
Constant	5.079***	4.244***	4.743***
	(18.28)	(11.81)	(13.70)
N	116	116	116
R^2^	0.900	0.932	0.846
adj.R^2^	0.894	0.928	0.836

EPU, GPR, CPU, PSE, CSI, FX, and DR represent economic policy uncertainty, geopolitical risk, climate policy uncertainty, Arca Tech 100 Index, CSI 300 index, the exchange rate of US dollar to Chinese yuan, and a dummy variable for crisis periods, respectively. The “Total” column represents the OLS regression results of the variable on the total spillover effect, while the “Positive” and “Negative” columns respectively represent the regression results of the variable on positive and negative spillovers.

We also verify the effect of uncertainties on asymmetric spillovers, that is, on positive and negative spillovers. The regression results for positive spillovers show that in the ‘positive’ column, among the various types of uncertainty, climate policy uncertainty has a remarkable inverse effect on positive spillovers, whereas economic policy uncertainty and global geopolitical uncertainty do not have a significant effect on positive spillovers. The variables (PSE, CSI, FX) give a significant effect on positive total connectedness, with PSE having a negative effect and CSI and FX having a positive effect. DR, being a dummy variable for the crisis period, does not give a significant effect on positive spillovers. An examination of the negative spillovers, as reflected in the “negative” column, reveals that the significance of the variables is basically the same as the total spillovers, i.e., EPU, CSI, FX, and DR positively affect negative spillovers, and CPU and PSE have a negative correlation on negative spillovers. In general, among the various uncertainties, only climate policy uncertainty has a notable impact on all three types of spillovers. Economic policy uncertainty has a significant effect on both total and negative spillovers, while geopolitical risk does not have a significant effect on any of the three spillovers. That is, both investors and policymakers should pay attention to changes in climate policy uncertainty as well as economic policy uncertainty because they can cause some degree of shock to the market. Moreover, during bad news shocks, economic uncertainty and climate policy uncertainty are sensitive to spillovers, while during good news shocks, only climate policy uncertainty is sensitive to spillovers. Therefore, the attitude towards these two types of uncertainty should be different under different shocks.

### 4.4 Robust testing

We estimate the robustness of inter-market asymmetric spillovers in the time-frequency domain for different window periods as well as the robustness of the regression relationship between uncertainties and spillovers. To ensure that the rolling windows do not interfere with the experiments, we set the rolling windows to two, one larger than 200 (250) and one smaller than 200 (150), and the experimental results are presented in Appendices A2-A24.After changing the window sizes, neither the static connectedness nor the dynamic connectedness nor the asymmetry challenge the original conclusions. The fact that the change in the rolling window does not alter the main conclusions is sufficient to demonstrate the robustness of asymmetric spillovers between markets. In addition, we regress the spillovers on uncertainty across different windows, and the regression results are shown in A23 and A24 for rolling windows of 150 and 250, respectively. Again, the significance and direction of the regression results do not change significantly, again demonstrating the robustness of the regression conclusions.

## 5. Conclusions

This paper contributes to the existing literature by using a combined time- and frequency-domain correlation approach to investigate the risk spillover relationship between clean energy markets and industrial equity markets. In this study, a risk spillover connectedness network is constructed using the DY model, and the BK model is used to analyse the performance of connectedness between markets in different frequency bands. The use of these two approaches not only reveals dynamic and static features across time and in different frequency domains, but also allows for a more comprehensive analysis of asymmetric spillover effects. While the static connectedness characteristics can represent the general situation, the dynamic connectedness characteristics take a time-varying perspective and consider the inter-market risk spillovers over time, especially the market performance during the periods of the four major events. The categorisation of spillovers into different frequency bands considers the ability of markets to process information. Asymmetric spillovers consider the effect of good and bad news on market spillovers. In addition, this paper explores the impact of various uncertainties on inter-market risk spillovers. The results of the study are summarised below.

The results of the static and dynamic spillovers between the clean energy market and the industrial stock market over the sample period show that there is a strong connectedness between the two markets. From a static connectedness perspective, it is evident that the industrial stock market is a net transmitter of risk, while the clean energy market is a net receiver of risk. This implies that the industrial stock market is the main source of risk in the systematic network, while the clean energy market is the receiver of risk. Specific to the sub-markets, the hydropower market outputs and receives the least amount of risk, indicating a relatively low degree of connectedness with other markets. In times of crisis, the hydropower market receives the most risk from the network, acting as the strongest risk receiver. The low-carbon portfolio has high connectedness to the network, and it exports the most risk as well as receives the most risk. In addition, the dynamic connectedness results show that the spillovers between the clean energy market and the industrial stock market are clearly time-varying, with the correlation between the two markets rising rapidly and the level of inter-market connectedness being high during major events, such as during the Chinese stock market crash, during the U.S.-China trade conflict, during COVID-19 contagion, and during the Russian-Ukrainian conflict. The greatest increase in inter-market connectedness occurred during the Russian-Ukrainian conflict, indicating that turbulence in neighbouring countries has a significant impact on the markets. It is also important to emphasise that both the static and dynamic correlation results indicate the need to focus on the low-carbon portfolio, which is highly contagious and a contributor to risk throughout the sample period. The results for the frequency bands show an absolute leadership of risk spillovers in the high frequency bands, indicating that the clean energy market and the industrial stock market exchanged information very quickly, transmitting most of the shocks to all assets within a week. In addition, the results on asymmetric risk spillovers suggest that bad news shocks have a greater impact on the network formed by the two markets, a finding that also holds in the high frequency band, but in the low frequency band, good news shocks cause stronger risk spillovers than bad news. This also suggests that in the short run bad news shocks are larger for the market, but good news shocks have a relatively longer-lasting effect on the market. Finally, the results of the effect of uncertainty on spillovers show that economic uncertainty and climate uncertainty have a significant effect on spillovers. Where a rise in economic uncertainty tends to lead to a rise in market connectedness, conversely, a rise in climate policy uncertainty can lead to a rise in market-to-self risk spillovers.

The results of this study provide practical guidance for investors to realise the benefits of diversification. Our overriding recommendation to investors is to optimise their portfolios by adjusting sectoral portfolio weights based on connectedness indicators. In particular, the hydro market is an effective hedging instrument, especially in times of crisis, as it is the strongest net risk receiver, bearing the risk spillover from other markets to it. It is worth emphasising that it is not a good choice to invest in a mix of low carbon portfolios, normal portfolios and high carbon portfolios, as the high correlation between the three assets may reduce the efficiency of the portfolios. When economic uncertainty rises, hedging assets should be strengthened to avoid unnecessary losses due to resonance effects between market segments. When climate policy uncertainty rises, it may not be a good idea to invest in only one sector, as it can lead to risk spillovers from the sector itself to itself, which can easily lead to larger losses. In addition, we also suggest that investors should time their investments through correlation results. The low carbon portfolio is the centre of the whole network, exporting the greatest risk and receiving the greatest risk. Therefore, a low-carbon portfolio can act as a weathervane, and when it starts to fluctuate dramatically, a brief exit from the stock market is a better option. Price linkages across all assets are exacerbated when a sudden major event occurs, so it is wise to avoid investing in times of major events. Categorising investors into long-term and short-term investors, for short-term investors, one should try to reduce their positions when bad news comes and keep an eye on the market and re-enter when the market starts to plateau. For long-term investors, it may be wise to increase a portion of your investments when good news occurs, as the impact of good news will be more lasting and may lead to more gains over time.

For policymakers, our research provides some useful insights for stabilising financial markets. Policymakers need to clarify the relationship between clean energy markets and industrial equity markets and understand the complex risk spillover dynamics between these two markets. Our most important recommendation to managers is to establish a real-time detection system for spillovers. It is important to monitor changes in the low-carbon sector to avoid it spilling risks to other markets. Especially when going through a period of crisis, failure to regulate the main spillover sectors will significantly disrupt other markets and trigger a financial crisis. Industrial equity markets are risk senders that can exert a lot of influence on clean energy markets. And the low-carbon sector is the one that should be most concerned, as it is the most powerful risk sender. In order to regulate changes in the low-carbon sector, regulators should give special regulation to sectors such as pharmaceuticals and electronics to stabilise market expectations. They should also take special measures to stabilise low-carbon portfolios when they detect sharp fluctuations in low-carbon markets, so that they can have a stabilising effect on all markets. It is important to emphasise that regulators need to pay attention to inter-market risk spillovers triggered by bad news, be vigilant in the event of unfavourable news in the market, proactively guide investors, and strengthen policy regulation to prevent shocks from being further amplified. Another recommendation we have for policymakers is to increase policy transparency and give markets a clear picture of what to expect. A rise in economic policy uncertainty is extremely likely to lead to a rise in market connectedness and enhance inter-market risk spillovers, which is not conducive to market stability. When an economic policy is enacted, it is desirable to provide a clear interpretation of the policy to increase policy transparency.

This paper has some limitations in its methodology and use of data. The methodology used in this paper to identify spillovers is the traditional TVP-VAR model, and it is possible that the use of a more cutting-edge spillover identification model could provide a more robust account as spillover identification models evolve. Additionally, this paper divided the frequency bands into one week and more than one week, and perhaps future research could subdivide the frequency bands again into one day, two to five days, and more than five days. Future research could also look at other types of uncertainty or apply the analysis to different regions or time periods.

## Supporting information

S1 File Original data, code, and appendix content.
(ZIP)
