# Supplementary material for: Asymmetric spillover connectedness between clean energy markets and industrial stock markets: How uncertainties affect it
Source: PLoS One. 2025 Mar 31;20(3):e0316171. doi: 10.1371/journal.pone.0316171 (PMC11957356; doi:10.1371/journal.pone.0316171)
Supplement: S1 File — Original data, code, and appendix content. (ZIP) [file pone.0316171.s001.zip › Supporting information/Appendix.docx]

**A 1. Portfolio type and average industry carbon emission.**

| **Portfolio Type** | **Code** | **CSRC Industry Name** | **Carbon Emission (Mt)** |
| --- | --- | --- | --- |
| Low-carbon Portfolio | C40 | Instruments, Meters, Cultural and Office Machinery | 0.31 |
|  | D46 | Production and Supply of Tap Water | 0.50 |
|  | C21 | Furniture Manufacturing | 0.61 |
|  | C23 | Printing and Record Medium Reproduction | 0.71 |
|  | C19 | Leather, Furs, Down and Related Products | 1.63 |
|  | C39 | Electronic and Telecommunications Equipment | 1.63 |
|  | B09 | Nonferrous Metals Mining and Dressing | 1.89 |
|  | C37 | Transportation Equipment | 2.04 |
|  | C18 | Garments and Other Fiber Products | 2.85 |
|  | B07 | Petroleum and Natural Gas Extraction | 3.45 |
|  | C35 | Equipment for Special Purposes | 3.82 |
|  | B08 | Ferrous Metals Mining and Dressing | 3.99 |
| Ordinary Portfolio | C33 | Metal Products | 5.03 |
|  | C34 | Ordinary Machinery | 5.04 |
|  | C20 | Timber Processing, Bamboo, Cane, Palm Fiber & Straw Products | 5.47 |
|  | C36 | Automobile Manufacturing Industry | 5.62 |
|  | C38 | Electric Equipment and Machinery | 6.02 |
|  | C41 | Other Manufacturing Industry | 7.02 |
|  | D45 | Production and Supply of Gas | 8.15 |
|  | C29 | Rubber Products | 10.49 |
|  | C28 | Chemical Fiber | 11.16 |
|  | C15 | Beverage Production | 13.55 |
|  | C27 | Medical and Pharmaceutical Products | 13.59 |
|  | C14 | Food Production | 17.73 |
|  | C13 | Food Processing | 29.23 |
|  | C17 | Textile Industry | 34.76 |
|  | C22 | Papermaking and Paper Products | 49.98 |
| High-carbon Portfolio | C32 | Smelting and Pressing of Nonferrous Metals | 111.65 |
|  | C26 | Raw Chemical Materials and Chemical Products | 259.16 |
|  | B06 | Coal Mining and Dressing | 304.66 |
|  | C30 | Nonmetal Mineral Products | 311.07 |
|  | C31 | Smelting and Pressing of Ferrous Metals | 326.42 |
|  | C25 | Petroleum Processing and Coking | 975.24 |
|  | D44 | Production and Supply of Electric Power, Steam and Hot Water | 1727.31 |

Notes: The industry is based on the industry classification standard of the China Securities Regulatory Commission (CSRC). The average industry carbon emissions are calculated based on the emission from 2010 to 2017 (Unit: Million Ton) by China Energy Statistical Yearbook.

**A 2. Static connectedness with a rolling window of 150**

|  | HY | WP | SP | LC | OR | HC | FROM |
| --- | --- | --- | --- | --- | --- | --- | --- |
| HY | 52.52 | 7.35 | 6.48 | 10.41 | 10.49 | 12.74 | 47.48 |
|  | **47.61** | **6.56** | **5.85** | **9.39** | **9.45** | **11.46** | **42.71** |
|  | ***4.91*** | ***0.8*** | ***0.63*** | ***1.02*** | ***1.04*** | ***1.28*** | ***4.77*** |
| WP | 5.17 | 31.41 | 15.66 | 16.63 | 15.65 | 15.48 | 68.59 |
|  | **4.66** | **28.44** | **14.09** | **15.09** | **14.11** | **14.01** | **61.95** |
|  | ***0.52*** | ***2.97*** | ***1.57*** | ***1.53*** | ***1.55*** | ***1.47*** | ***6.64*** |
| SP | 4.64 | 15.67 | 30.94 | 16.28 | 16.86 | 15.61 | 69.06 |
|  | **4.17** | **14.22** | **27.95** | **14.82** | **15.16** | **14.2** | **62.57** |
|  | ***0.47*** | ***1.46*** | ***2.99*** | ***1.46*** | ***1.69*** | ***1.41*** | ***6.5*** |
| LC | 6.1 | 13.96 | 13.74 | 25.66 | 19.35 | 21.19 | 74.34 |
|  | **5.46** | **12.57** | **12.3** | **23.13** | **17.29** | **19.03** | **66.66** |
|  | ***0.64*** | ***1.39*** | ***1.44*** | ***2.53*** | ***2.05*** | ***2.16*** | ***7.69*** |
| OR | 6.22 | 13.71 | 14.8 | 20.23 | 27.33 | 17.71 | 72.67 |
|  | **5.64** | **12.44** | **13.43** | **18.4** | **24.67** | **16.05** | **65.96** |
|  | ***0.58*** | ***1.27*** | ***1.37*** | ***1.83*** | ***2.66*** | ***1.66*** | ***6.71*** |
| HC | 7.44 | 13.37 | 13.52 | 21.79 | 17.59 | 26.29 | 73.71 |
|  | **6.69** | **12.11** | **12.19** | **19.74** | **15.81** | **23.74** | **66.54** |
|  | ***0.75*** | ***1.26*** | ***1.33*** | ***2.05*** | ***1.78*** | ***2.55*** | ***7.17*** |
| TO | 29.57 | 64.07 | 64.21 | 85.34 | 79.94 | 82.72 |  |
|  | **26.61** | **57.89** | **57.87** | **77.44** | **71.82** | **74.75** |  |
|  | ***2.96*** | ***6.18*** | ***6.34*** | ***7.9*** | ***8.11*** | ***7.98*** | TCI |
| Net | -17.91 | -4.52 | -4.85 | 11 | 7.27 | 9.01 | 67.64 |
|  | **-16.1** | **-4.06** | **-4.7** | **10.79** | **5.87** | **8.21** | **61.06** |
|  | ***-1.81*** | ***-0.46*** | ***-0.15*** | ***0.21*** | ***1.4*** | ***0.81*** | ***6.58*** |

Note: HY, WP, SP, LC, OR, and HC represent the hydropower market, wind power market, solar power market, low-carbon industrial market, ordinary industrial market, and high-carbon industrial market, respectively. Normal font represents total spillover levels, bold represents short-term spillover levels, and italic represents long-term spillover levels.

**A 3. Asymmetric static connectedness in the full frequency band with a rolling window of 150**

|  | HY | WP | SP | LC | OR | HC | FROM |
| --- | --- | --- | --- | --- | --- | --- | --- |
| HY | 60.61 | 6.05 | 5.48 | 8.74 | 8.61 | 10.51 | 39.39 |
|  | (46.21) | (8.71) | (8.4) | (11.2) | (11.98) | (13.49) | (53.79) |
| WP | 4.75 | 38.22 | 15.59 | 14.44 | 14.18 | 12.82 | 61.78 |
|  | (6.48) | (30.25) | (15.13) | (16.84) | (15.49) | (15.82) | (69.75) |
| SP | 4.24 | 14.98 | 37.52 | 14.59 | 14.95 | 13.73 | 62.48 |
|  | (6.35) | (15.4) | (31.18) | (15.67) | (16.51) | (14.9) | (68.82) |
| LC | 5.27 | 11.37 | 11.82 | 29.66 | 20.42 | 21.47 | 70.34 |
|  | (7.33) | (14.57) | (13.55) | (25.23) | (18.58) | (20.74) | (74.77) |
| OR | 5.18 | 11.57 | 12.6 | 21.45 | 31.68 | 17.51 | 68.32 |
|  | (7.81) | (13.93) | (14.67) | (19.23) | (27.11) | (17.26) | (72.89) |
| HC | 6.5 | 10.69 | 11.63 | 22.5 | 17.65 | 31.03 | 68.97 |
|  | (8.77) | (13.94) | (13.16) | (21.16) | (17.15) | (25.83) | (74.17) |
| TO | 25.94 | 54.65 | 57.13 | 81.72 | 75.81 | 76.03 |  |
|  | (36.74) | (66.54) | (64.91) | (84.1) | (79.7) | (82.2) | TCI |
| Net | -13.45 | -7.12 | -5.35 | 11.38 | 7.49 | 7.06 | 61.88 |
|  | (-17.04) | (-3.21) | (-3.91) | (9.33) | (6.81) | (8.03) | (69.03) |

Note: HY, WP, SP, LC, OR, and HC represent the hydropower market, wind power market, solar power market, low-carbon industrial market, ordinary industrial market, and high-carbon industrial market, respectively. Values inside parentheses are negative spillovers, values without parentheses represent positive spillovers.

**A 4. Asymmetric static connectedness in the high frequency band with a rolling window of 150**

|  | HY | WP | SP | LC | OR | HC | FROM |
| --- | --- | --- | --- | --- | --- | --- | --- |
| HY | 53.13 | 4.87 | 4.22 | 7.13 | 7.01 | 8.78 | 32 |
|  | (40.95) | (7.44) | (7.2) | (9.75) | (10.39) | (11.8) | (46.57) |
| WP | 3.83 | 33.59 | 13.15 | 12.33 | 12.08 | 11.12 | 52.52 |
|  | (5.52) | (26.99) | (13.26) | (14.94) | (13.59) | (13.98) | (61.29) |
| SP | 3.26 | 12.86 | 32.65 | 12.3 | 12.57 | 11.8 | 52.79 |
|  | (5.31) | (13.5) | (27.6) | (13.88) | (14.48) | (13.19) | (60.36) |
| LC | 4.26 | 9.78 | 9.99 | 25.85 | 17.57 | 18.69 | 60.29 |
|  | (6.27) | (12.76) | (11.87) | (22.35) | (16.27) | (18.3) | (65.48) |
| OR | 4.25 | 10.02 | 10.8 | 18.68 | 27.82 | 15.24 | 58.99 |
|  | (6.73) | (12.26) | (12.96) | (17.1) | (24.04) | (15.3) | (64.36) |
| HC | 5.38 | 9.33 | 10.02 | 19.67 | 15.3 | 27.25 | 59.7 |
|  | (7.58) | (12.27) | (11.56) | (18.82) | (15.09) | (22.96) | (65.31) |
| TO | 20.99 | 46.86 | 48.19 | 70.1 | 64.53 | 65.63 |  |
|  | (31.42) | (58.24) | (56.85) | (74.48) | (69.82) | (72.57) | TCI |
| Net | -11.01 | -5.66 | -4.6 | 9.81 | 5.53 | 5.93 | 52.71 |
|  | (-15.16) | (-3.06) | (-3.5) | (9) | (5.46) | (7.26) | (60.56) |

Note: HY, WP, SP, LC, OR, and HC represent the hydropower market, wind power market, solar power market, low-carbon industrial market, ordinary industrial market, and high-carbon industrial market, respectively. Values inside parentheses are negative spillovers, values without parentheses represent positive spillovers.

**A 5. Asymmetric static connectedness in the low frequency band with a rolling window of 150**

|  | HY | WP | SP | LC | OR | HC | FROM |
| --- | --- | --- | --- | --- | --- | --- | --- |
| HY | 7.48 | 1.17 | 1.27 | 1.62 | 1.6 | 1.73 | 7.39 |
|  | (5.27) | (1.27) | (1.2) | (1.46) | (1.58) | (1.7) | (7.21) |
| WP | 0.91 | 4.63 | 2.43 | 2.12 | 2.1 | 1.7 | 9.26 |
|  | (0.96) | (3.26) | (1.87) | (1.91) | (1.9) | (1.83) | (8.46) |
| SP | 0.97 | 2.12 | 4.86 | 2.29 | 2.39 | 1.93 | 9.69 |
|  | (1.03) | (1.9) | (3.58) | (1.79) | (2.03) | (1.71) | (8.46) |
| LC | 1.01 | 1.59 | 1.83 | 3.81 | 2.85 | 2.78 | 10.06 |
|  | (1.06) | (1.8) | (1.68) | (2.88) | (2.31) | (2.44) | (9.29) |
| OR | 0.93 | 1.55 | 1.8 | 2.77 | 3.86 | 2.27 | 9.32 |
|  | (1.08) | (1.66) | (1.7) | (2.12) | (3.07) | (1.96) | (8.53) |
| HC | 1.12 | 1.36 | 1.61 | 2.83 | 2.35 | 3.78 | 9.27 |
|  | (1.19) | (1.67) | (1.6) | (2.34) | (2.06) | (2.87) | (8.87) |
| TO | 4.95 | 7.8 | 8.94 | 11.62 | 11.28 | 10.41 |  |
|  | (5.33) | (8.31) | (8.05) | (9.62) | (9.88) | (9.64) | TCI |
| Net | -2.44 | -1.46 | -0.75 | 1.56 | 1.96 | 1.13 | 9.17 |
|  | (-1.89) | (-0.16) | (-0.41) | (0.33) | (1.35) | (0.77) | (8.47) |

Note: HY, WP, SP, LC, OR, and HC represent the hydropower market, wind power market, solar power market, low-carbon industrial market, ordinary industrial market, and high-carbon industrial market, respectively. Values inside parentheses are negative spillovers, values without parentheses represent positive spillovers.

**A 6. Static connectedness with a rolling window of 250**

|  | HY | WP | SP | LC | OR | HC | FROM |
| --- | --- | --- | --- | --- | --- | --- | --- |
| HY | 52.54 | 7.23 | 6.48 | 10.51 | 10.43 | 12.8 | 47.46 |
|  | **47.69** | **6.45** | **5.85** | **9.49** | **9.4** | **11.54** | **42.74** |
|  | ***4.85*** | ***0.78*** | ***0.63*** | ***1.02*** | ***1.03*** | ***1.27*** | ***4.72*** |
| WP | 5.25 | 31.62 | 15.65 | 16.53 | 15.62 | 15.34 | 68.38 |
|  | **4.69** | **28.56** | **14.05** | **14.96** | **14.04** | **13.85** | **61.6** |
|  | ***0.55*** | ***3.05*** | ***1.6*** | ***1.57*** | ***1.58*** | ***1.48*** | ***6.79*** |
| SP | 4.73 | 15.6 | 31.07 | 16.23 | 16.86 | 15.52 | 68.93 |
|  | **4.22** | **14.09** | **28.03** | **14.71** | **15.12** | **14.09** | **62.23** |
|  | ***0.51*** | ***1.51*** | ***3.04*** | ***1.52*** | ***1.74*** | ***1.43*** | ***6.7*** |
| LC | 6.27 | 13.78 | 13.67 | 25.72 | 19.33 | 21.23 | 74.28 |
|  | **5.58** | **12.36** | **12.22** | **23.14** | **17.25** | **19.04** | **66.44** |
|  | ***0.69*** | ***1.42*** | ***1.46*** | ***2.58*** | ***2.09*** | ***2.19*** | ***7.84*** |
| OR | 6.27 | 13.59 | 14.81 | 20.22 | 27.41 | 17.7 | 72.59 |
|  | **5.66** | **12.28** | **13.42** | **18.35** | **24.71** | **16.02** | **65.73** |
|  | ***0.61*** | ***1.31*** | ***1.4*** | ***1.87*** | ***2.7*** | ***1.68*** | ***6.85*** |
| HC | 7.6 | 13.16 | 13.43 | 21.82 | 17.56 | 26.43 | 73.57 |
|  | **6.81** | **11.89** | **12.09** | **19.74** | **15.76** | **23.84** | **66.29** |
|  | ***0.79*** | ***1.27*** | ***1.34*** | ***2.08*** | ***1.8*** | ***2.58*** | ***7.28*** |
| TO | 30.12 | 63.35 | 64.05 | 85.31 | 79.8 | 82.58 |  |
|  | **26.97** | **57.07** | **57.63** | **77.26** | **71.57** | **74.53** |  |
|  | ***3.15*** | ***6.28*** | ***6.42*** | ***8.05*** | ***8.23*** | ***8.05*** | TCI |
| Net | -17.34 | -5.03 | -4.89 | 11.03 | 7.22 | 9.01 | 67.54 |
|  | **-15.76** | **-4.53** | **-4.61** | **10.82** | **5.84** | **8.24** | **60.84** |
|  | ***-1.58*** | ***-0.51*** | ***-0.28*** | ***0.22*** | ***1.38*** | ***0.77*** | ***6.70*** |

Note: HY, WP, SP, LC, OR, and HC represent the hydropower market, wind power market, solar power market, low-carbon industrial market, ordinary industrial market, and high-carbon industrial market, respectively. Normal font represents total spillover levels, bold represents short-term spillover levels, and italic represents long-term spillover levels.

**A 7. Asymmetric static connectedness in the full frequency band with a rolling window of 250**

|  | HY | WP | SP | LC | OR | HC | FROM |
| --- | --- | --- | --- | --- | --- | --- | --- |
| HY | 60.51 | 6 | 5.52 | 8.93 | 8.65 | 10.4 | 39.49 |
|  | (46.54) | (8.52) | (8.33) | (11.25) | (11.83) | (13.54) | (53.46) |
| WP | 4.83 | 38.18 | 15.52 | 14.39 | 14.19 | 12.89 | 61.82 |
|  | (6.31) | (30.34) | (15.19) | (16.83) | (15.55) | (15.77) | (69.66) |
| SP | 4.38 | 14.86 | 37.47 | 14.6 | 14.97 | 13.71 | 62.53 |
|  | (6.26) | (15.42) | (31.2) | (15.68) | (16.55) | (14.89) | (68.8) |
| LC | 5.33 | 11.26 | 11.79 | 29.66 | 20.42 | 21.53 | 70.34 |
|  | (7.29) | (14.53) | (13.58) | (25.28) | (18.57) | (20.73) | (74.72) |
| OR | 5.24 | 11.52 | 12.59 | 21.47 | 31.71 | 17.48 | 68.29 |
|  | (7.65) | (13.97) | (14.73) | (19.24) | (27.17) | (17.24) | (72.83) |
| HC | 6.43 | 10.69 | 11.63 | 22.63 | 17.63 | 30.99 | 69.01 |
|  | (8.72) | (13.89) | (13.17) | (21.17) | (17.15) | (25.9) | (74.1) |
| TO | 26.21 | 54.33 | 57.05 | 82.02 | 75.86 | 76 |  |
|  | (36.24) | (66.34) | (65) | (84.17) | (79.65) | (82.17) |  |
| Net | -13.28 | -7.49 | -5.48 | 11.68 | 7.57 | 6.99 | 61.91 |
|  | (-17.22) | (-3.33) | (-3.8) | (9.45) | (6.82) | (8.07) | (68.93) |

Note: HY, WP, SP, LC, OR, and HC represent the hydropower market, wind power market, solar power market, low-carbon industrial market, ordinary industrial market, and high-carbon industrial market, respectively. Values inside parentheses are negative spillovers, values without parentheses represent positive spillovers.

**A 8. Asymmetric static connectedness in the high frequency band with a rolling window of 250**

|  | HY | WP | SP | LC | OR | HC | FROM |
| --- | --- | --- | --- | --- | --- | --- | --- |
| HY | 53.24 | 4.83 | 4.24 | 7.29 | 7.06 | 8.78 | 32.2 |
|  | (41.26) | (7.28) | (7.14) | (9.79) | (10.27) | (11.84) | (46.32) |
| WP | 3.91 | 33.53 | 13.07 | 12.26 | 12.06 | 11.18 | 52.48 |
|  | (5.37) | (27.07) | (13.32) | (14.93) | (13.65) | (13.94) | (61.21) |
| SP | 3.4 | 12.74 | 32.58 | 12.29 | 12.56 | 11.78 | 52.76 |
|  | (5.24) | (13.52) | (27.61) | (13.89) | (14.51) | (13.18) | (60.34) |
| LC | 4.35 | 9.67 | 9.95 | 25.83 | 17.56 | 18.76 | 60.29 |
|  | (6.24) | (12.74) | (11.9) | (22.4) | (16.26) | (18.3) | (65.44) |
| OR | 4.33 | 9.95 | 10.76 | 18.67 | 27.82 | 15.22 | 58.94 |
|  | (6.59) | (12.31) | (13.02) | (17.11) | (24.09) | (15.28) | (64.31) |
| HC | 5.39 | 9.32 | 10.01 | 19.76 | 15.28 | 27.26 | 59.76 |
|  | (7.54) | (12.23) | (11.57) | (18.83) | (15.08) | (23.02) | (65.25) |
| TO | 21.39 | 46.51 | 48.04 | 70.27 | 64.51 | 65.72 |  |
|  | (30.98) | (58.08) | (56.95) | (74.55) | (69.78) | (72.54) |  |
| Net | -10.81 | -5.97 | -4.72 | 9.98 | 5.57 | 5.96 | 52.74 |
|  | (-15.35) | (-3.13) | (-3.39) | (9.12) | (5.47) | (7.29) | (60.48) |

Note: HY, WP, SP, LC, OR, and HC represent the hydropower market, wind power market, solar power market, low-carbon industrial market, ordinary industrial market, and high-carbon industrial market, respectively. Values inside parentheses are negative spillovers, values without parentheses represent positive spillovers.

**A 9. Asymmetric static connectedness in the low frequency band with a rolling window of 250**

|  | HY | WP | SP | LC | OR | HC | FROM |
| --- | --- | --- | --- | --- | --- | --- | --- |
| HY | 7.27 | 1.16 | 1.27 | 1.64 | 1.59 | 1.62 | 7.29 |
|  | (5.28) | (1.24) | (1.19) | (1.46) | (1.56) | (1.69) | (7.14) |
| WP | 0.92 | 4.65 | 2.45 | 2.13 | 2.12 | 1.71 | 9.34 |
|  | (0.94) | (3.26) | (1.87) | (1.91) | (1.9) | (1.83) | (8.45) |
| SP | 0.98 | 2.12 | 4.89 | 2.31 | 2.41 | 1.93 | 9.76 |
|  | (1.02) | (1.9) | (3.59) | (1.79) | (2.03) | (1.71) | (8.46) |
| LC | 0.98 | 1.6 | 1.84 | 3.83 | 2.86 | 2.77 | 10.05 |
|  | (1.05) | (1.8) | (1.69) | (2.88) | (2.31) | (2.44) | (9.28) |
| OR | 0.9 | 1.57 | 1.82 | 2.8 | 3.89 | 2.25 | 9.35 |
|  | (1.06) | (1.66) | (1.71) | (2.12) | (3.08) | (1.96) | (8.52) |
| HC | 1.04 | 1.37 | 1.62 | 2.87 | 2.35 | 3.73 | 9.25 |
|  | (1.18) | (1.66) | (1.6) | (2.34) | (2.06) | (2.88) | (8.85) |
| TO | 4.82 | 7.82 | 9.01 | 11.75 | 11.35 | 10.29 |  |
|  | (5.26) | (8.26) | (8.05) | (9.62) | (9.87) | (9.63) |  |
| Net | -2.48 | -1.51 | -0.75 | 1.7 | 2.01 | 1.04 | 9.17 |
|  | (-1.87) | (-0.19) | (-0.41) | (0.34) | (1.36) | (0.78) | (8.45) |

Note: HY, WP, SP, LC, OR, and HC represent the hydropower market, wind power market, solar power market, low-carbon industrial market, ordinary industrial market, and high-carbon industrial market, respectively. Values inside parentheses are negative spillovers, values without parentheses represent positive spillovers.


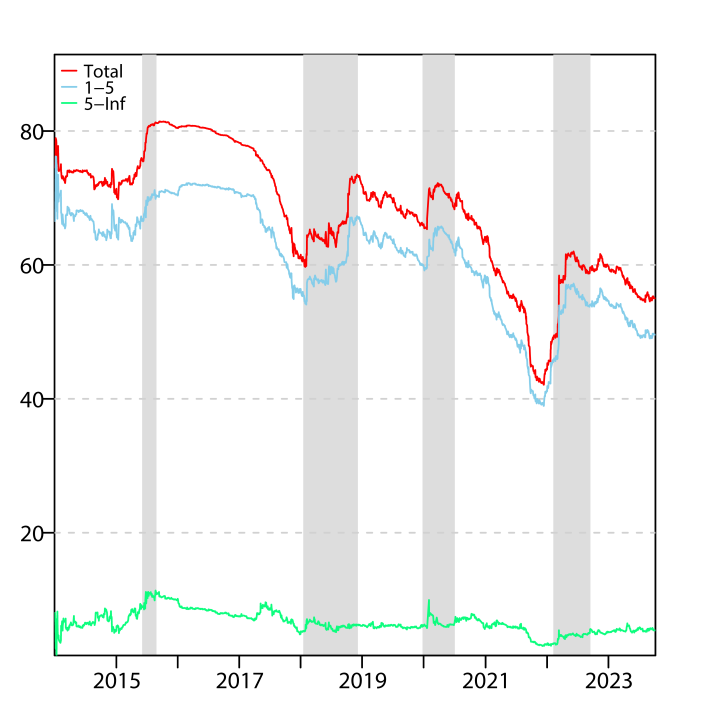


**A 11. Dynamic total spillover with a rolling window of 150**

Note: This figure shows the total spillovers between clean energy and industrial stock markets over time, especially during the four big events. The total spillovers reflect the level of connectedness between markets, with higher connectedness representing stronger price linkages between markets. The red line represents total spillover, the blue line represents short-term spillover (1-5 days), and the green line represents long-term spillover (more than 5 days).


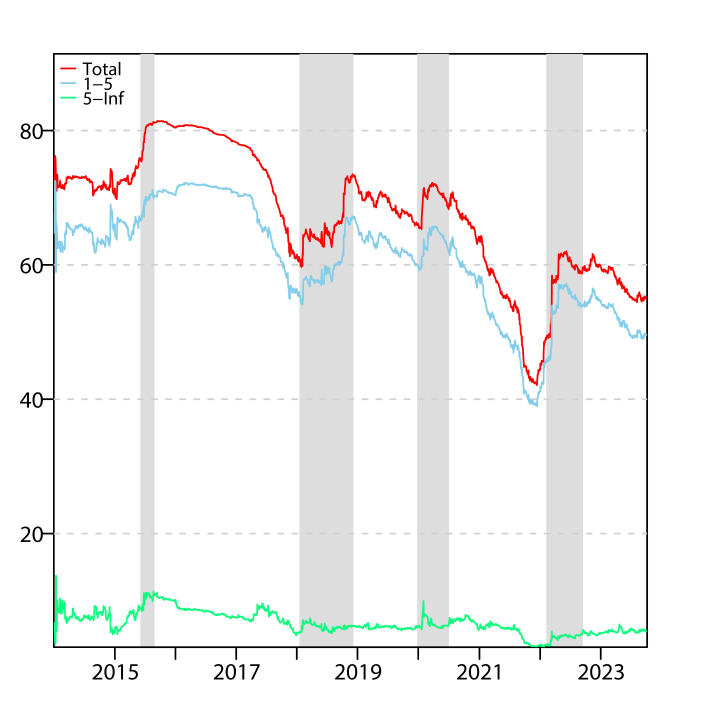


**A 12. Dynamic total spillover with a rolling window of 250**

Note: This figure shows the total spillovers between clean energy and industrial stock markets over time, especially during the four big events. The total spillovers reflect the level of connectedness between markets, with higher connectedness representing stronger price linkages between markets. The red line represents total spillover, the blue line represents short-term spillover (1-5 days), and the green line represents long-term spillover (more than 5 days).


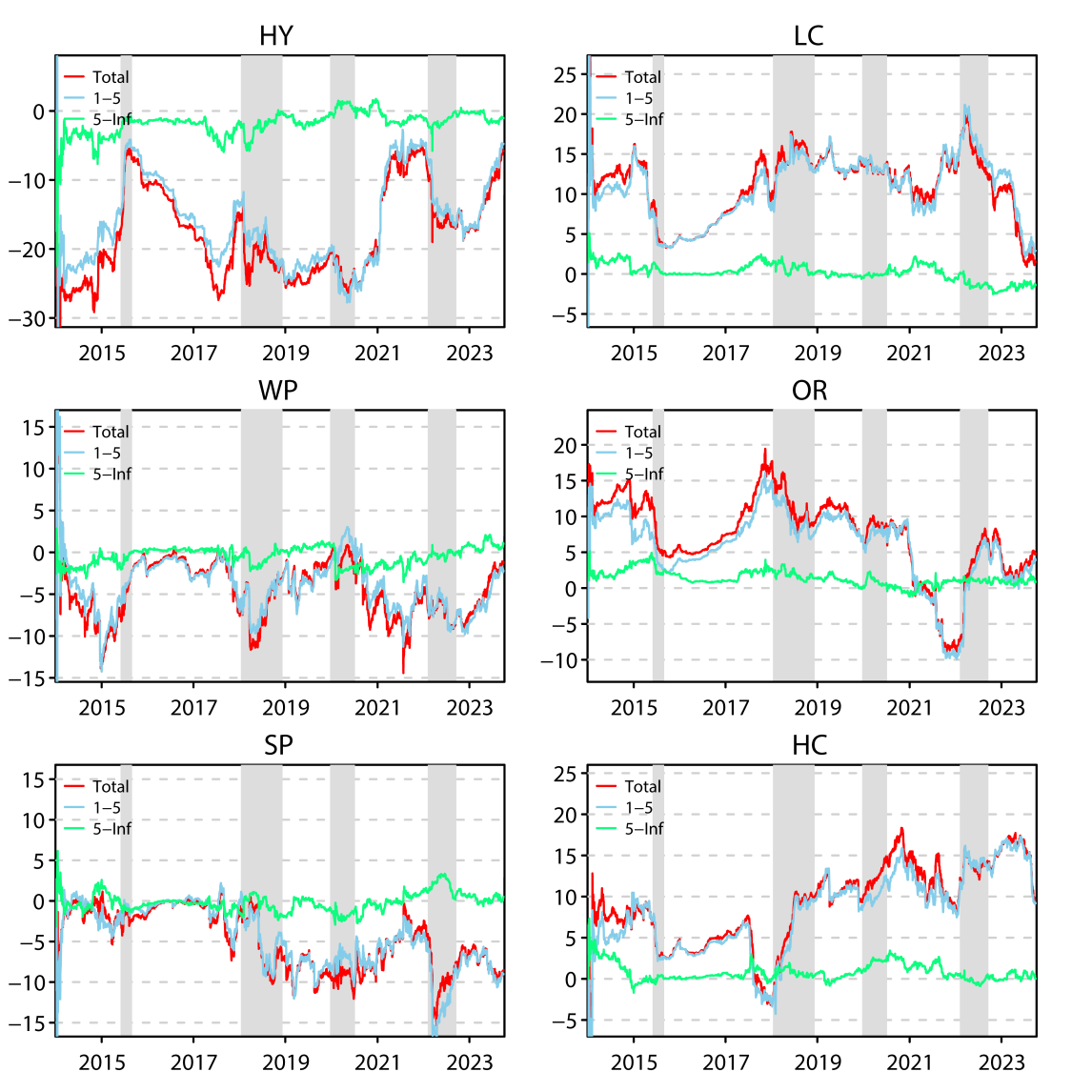


**A 13. Net return spillover with a rolling window of 150**

Note: This figure shows the difference between the influence that individual markets and networks exert on each other. Differences greater than 0 mean that the market mainly influences the network, while differences less than 0 mean that the market mainly absorbs influences from other markets. The red line represents total spillover, the blue line represents short-term spillover (1-5 days), and the green line represents long-term spillover (more than 5 days).


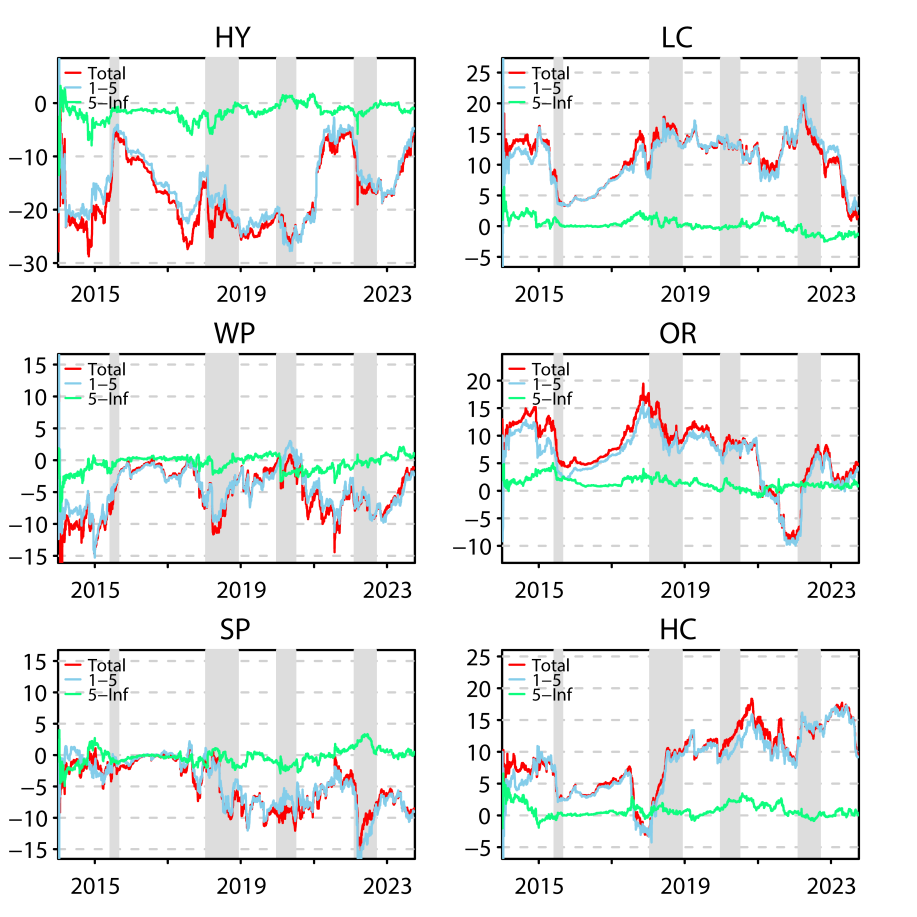


**A 14. Net return spillover with a rolling window of 250**

Note: This figure shows the difference between the influence that individual markets and networks exert on each other. Differences greater than 0 mean that the market mainly influences the network, while differences less than 0 mean that the market mainly absorbs influences from other markets. The red line represents total spillover, the blue line represents short-term spillover (1-5 days), and the green line represents long-term spillover (more than 5 days).


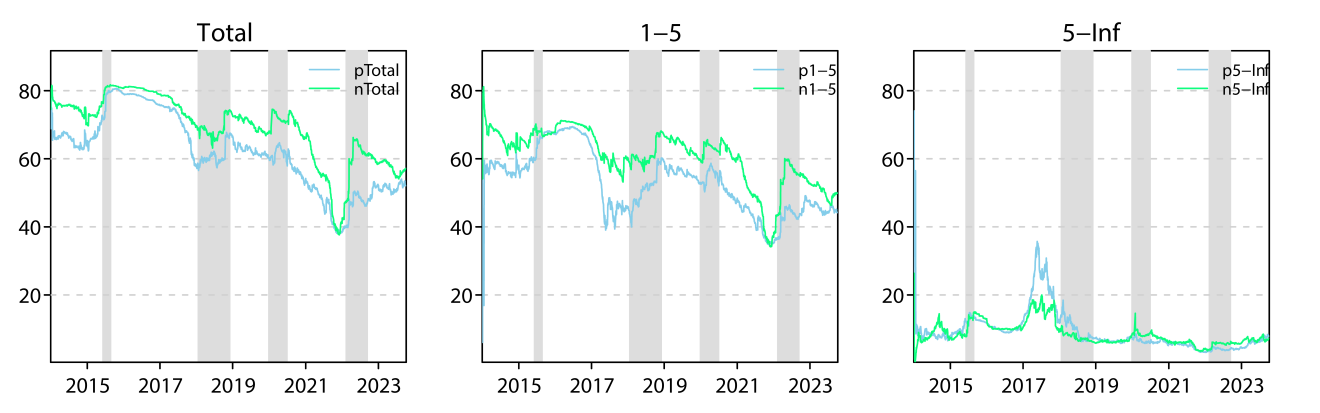


**A 15. Asymmetric dynamic total spillover in different frequency bands with a rolling window of 150**

Note: This figure shows spillovers between the clean energy and industrial stock markets at different frequency bands in response to good and bad news shocks. The blue line represents the positive total spillover index, and the green line represents the negative total spillover index. “1-5” refers to the short term, “5-Inf” refers to the long term, and their sum is “Total”.


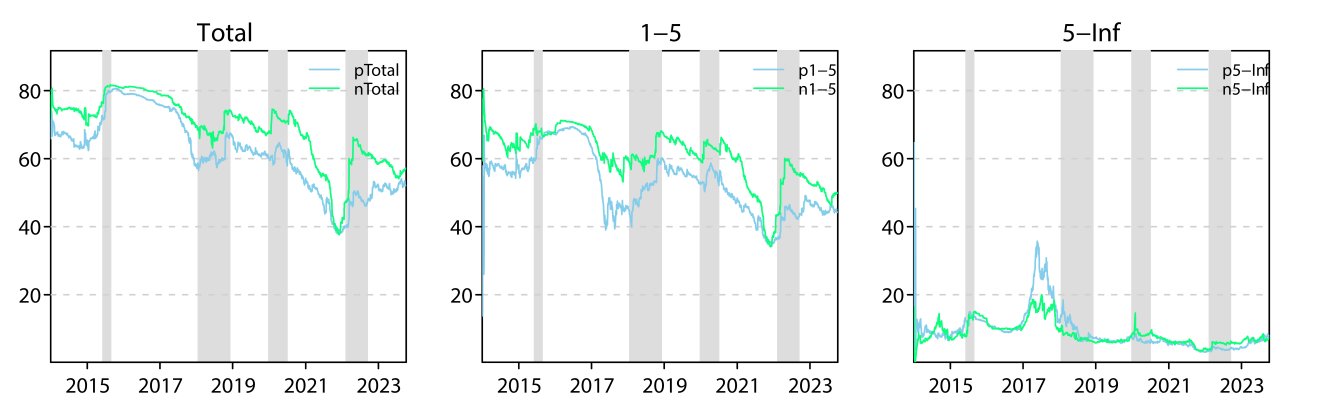


**A 16. Asymmetric dynamic total spillover in different frequency bands with a rolling window of 250**

Note: This figure shows spillovers between the clean energy and industrial stock markets at different frequency bands in response to good and bad news shocks. The blue line represents the positive total spillover index, and the green line represents the negative total spillover index. “1-5” refers to the short term, “5-Inf” refers to the long term, and their sum is “Total”.


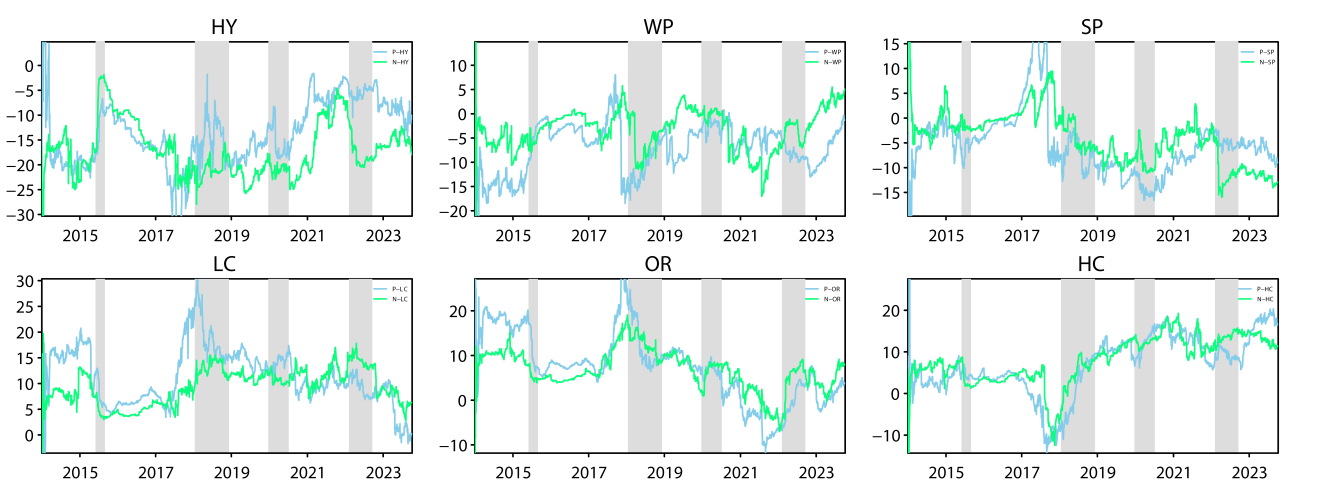


**A 17. Asymmetric net return spillovers in the full frequency band with a rolling window of 150**

Note: The figure shows the net spillovers across different markets in the full frequency band in response to good and bad news shocks. The blue line represents the positive net spillover index, and the green line represents the negative net spillover index.


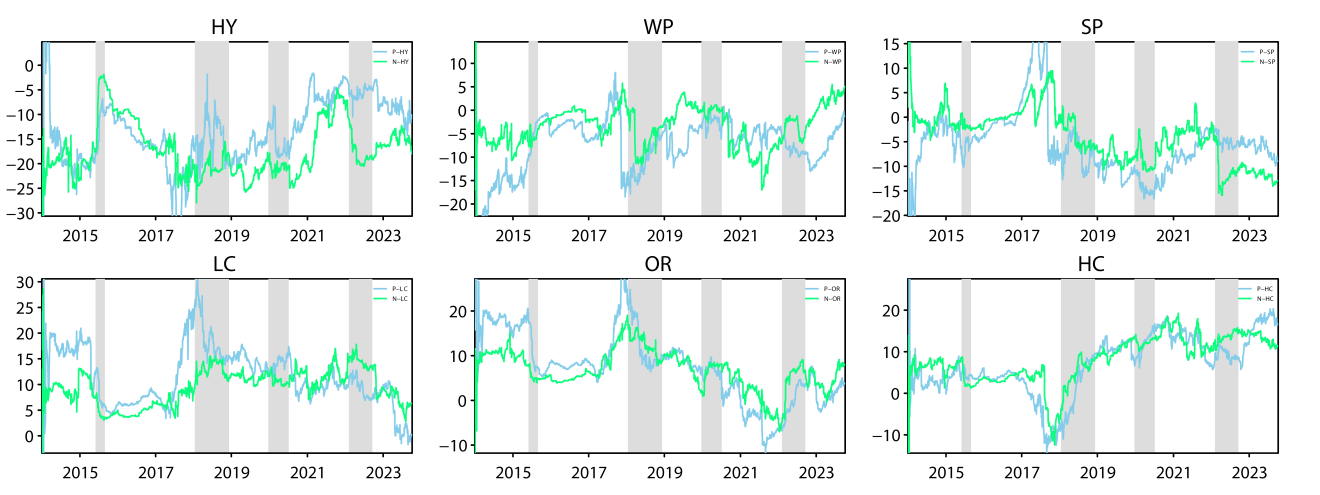


**A 18. Asymmetric net return spillovers in the full frequency band with a rolling window of 250**

Note: The figure shows the net spillovers across different markets in the full frequency band in response to good and bad news shocks. The blue line represents the positive net spillover index, and the green line represents the negative net spillover index.


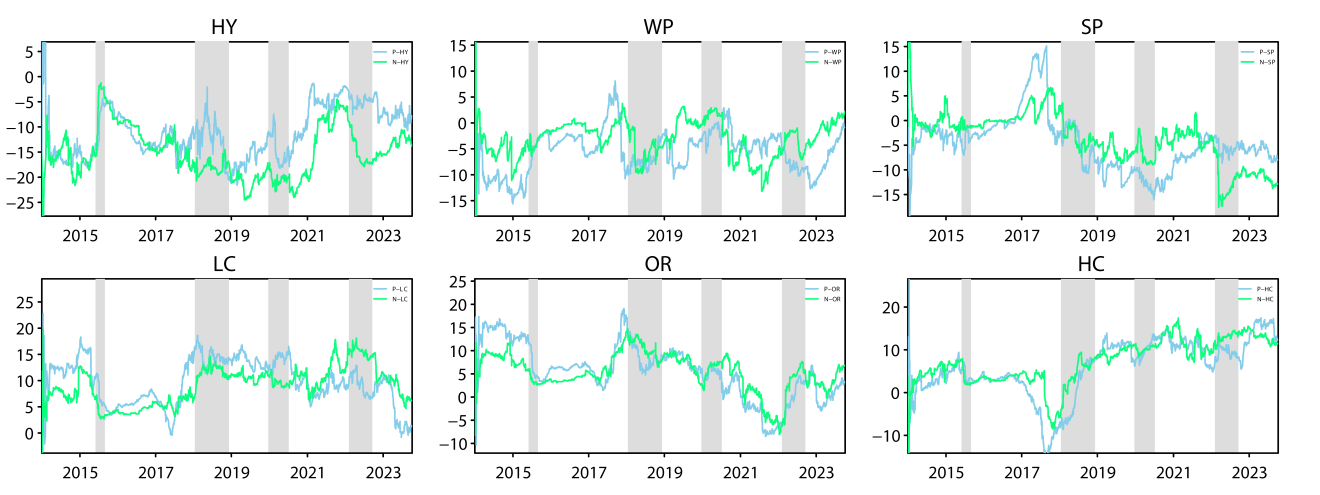


**A 19. Asymmetric dynamic return spillover in high frequency bands with a rolling window of 150**

Note: The figure illustrates the net spillover effect of a shock of good and bad news in a high frequency band. It shows the impact of good and bad news on the market in the short term. The blue line represents the positive net spillover index, and the green line represents the negative net spillover index.


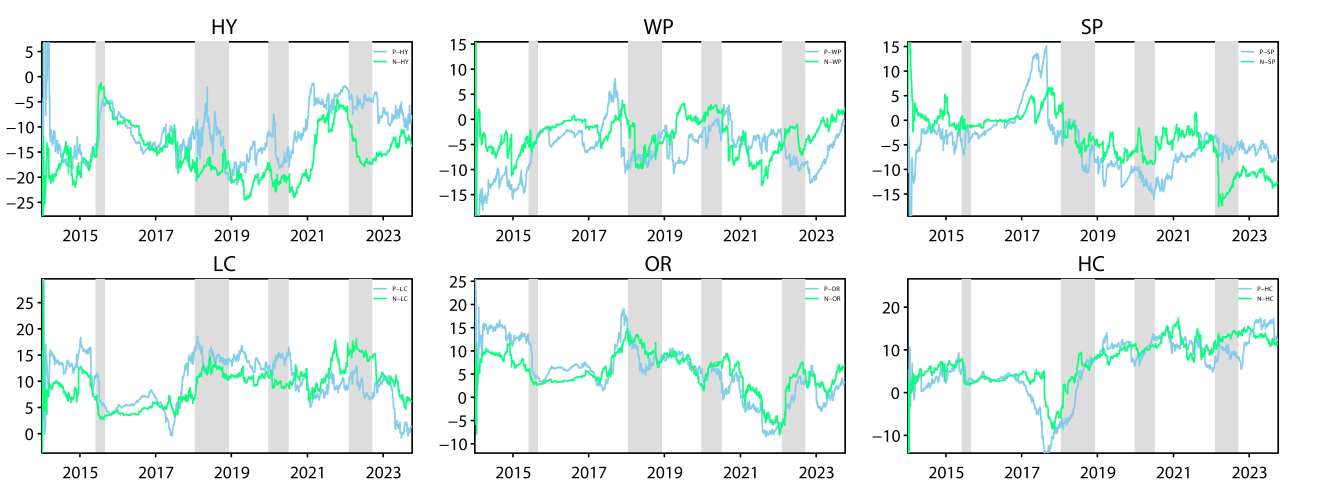


**A20. Asymmetric dynamic return spillover in high frequency bands with a rolling window of 250**

Note: The figure illustrates the net spillover effect of a shock of good and bad news in a high frequency band. It shows the impact of good and bad news on the market in the short term. The blue line represents the positive net spillover index, and the green line represents the negative net spillover index.


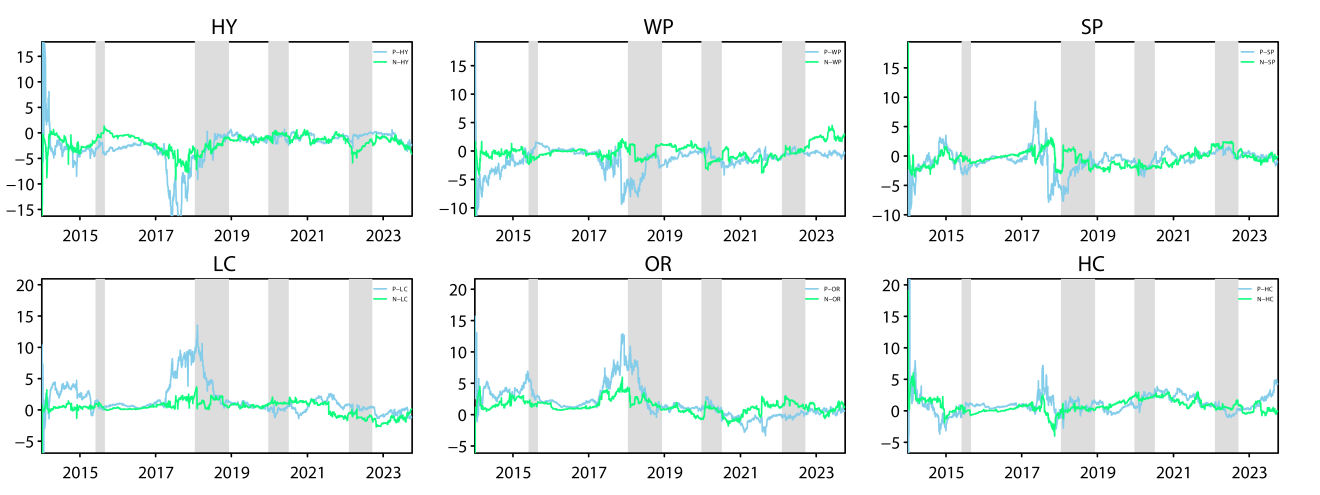


**A 21. Asymmetric dynamic return spillover in low frequency bands with a rolling window of 150**

Note: The graph shows the net spillover effects of good news and bad news shocks in a low frequency band. It shows the impact of good and bad news on the market in the long term. The blue line represents the positive net spillover index, and the green line represents the negative net spillover index.


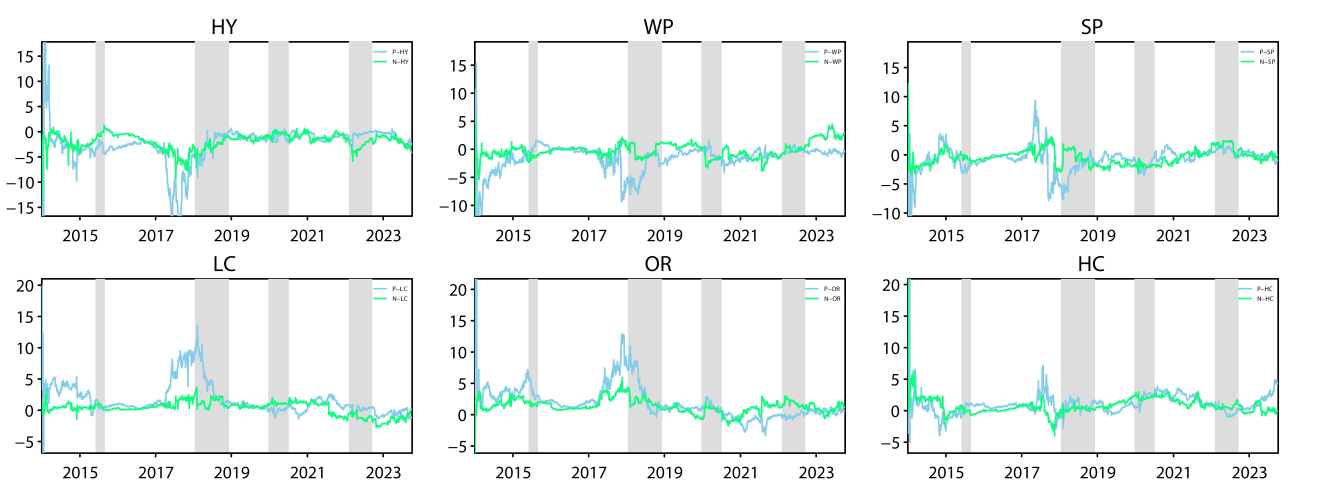


**A 22. Asymmetric dynamic return spillover in low frequency bands with a rolling window of 250**

Note: The graph shows the net spillover effects of good news and bad news shocks in a low frequency band. It shows the impact of good and bad news on the market in the long term. The blue line represents the positive net spillover index, and the green line represents the negative net spillover index.

**A 23. Regression results of uncertainties on spillover with a rolling window of 150**

|  | Total | Positive | Negative |
| --- | --- | --- | --- |
| EPU | 0.110*** | 0.0224 | 0.0960*** |
|  | (4.47) | (0.98) | (3.00) |
| GPR | -0.0286 | -0.0277 | -0.0474* |
|  | (-1.53) | (-1.33) | (-1.88) |
| CPU | -0.0461*** | -0.0373** | -0.0518** |
|  | (-2.91) | (-2.48) | (-2.23) |
| PSE | -0.457*** | -0.589*** | -0.467*** |
|  | (-24.43) | (-27.67) | (-18.46) |
| CSI | 0.111*** | 0.231*** | 0.121*** |
|  | (4.53) | (7.33) | (4.03) |
| FX | 0.824*** | 1.519*** | 0.942*** |
|  | (4.52) | (9.07) | (3.57) |
| DR | 0.0168* | 0.00380 | 0.0341** |
|  | (1.68) | (0.35) | (2.39) |
| Constant | 5.187*** | 4.261*** | 5.166*** |
|  | (18.16) | (11.84) | (14.15) |
| N | 116 | 116 | 116 |
| R^2^ | 0.900 | 0.932 | 0.845 |
| adj.R^2^ | 0.893 | 0.928 | 0.835 |

Notes: EPU, GPR, CPU, PSE, CSI, FX, and DR represent economic policy uncertainty, geopolitical risk, climate policy uncertainty, Arca Tech 100 Index, CSI 300 index, the exchange rate of US dollar to Chinese yuan, and a dummy variable for crisis periods, respectively. The “Total” column represents the OLS regression results of the variable on the total spillover effect, while the “Positive” and “Negative” columns respectively represent the regression results of the variable on positive and negative spillovers.

**A 24. Regression results of uncertainties on spillover with a rolling window of 250**

|  | Total | Positive | Negative |
| --- | --- | --- | --- |
| EPU | 0.110*** | 0.0227 | 0.0966*** |
|  | (4.52) | (1.00) | (3.05) |
| GPR | -0.0241 | -0.0295 | -0.0437* |
|  | (-1.30) | (-1.42) | (-1.75) |
| CPU | -0.0468*** | -0.0368** | -0.0524** |
|  | (-2.99) | (-2.44) | (-2.28) |
| PSE | -0.464*** | -0.587*** | -0.473*** |
|  | (-25.09) | (-27.59) | (-18.84) |
| CSI | 0.135*** | 0.224*** | 0.142*** |
|  | (5.61) | (7.10) | (4.80) |
| FX | 0.881*** | 1.497*** | 0.990*** |
|  | (4.90) | (8.92) | (3.79) |
| DR | 0.0175* | 0.00352 | 0.0348** |
|  | (1.77) | (0.32) | (2.44) |
| Constant | 4.922*** | 4.354*** | 4.933*** |
|  | (17.81) | (12.07) | (13.91) |
| N | 116 | 116 | 116 |
| R^2^ | 0.900 | 0.933 | 0.845 |
| adj.R^2^ | 0.894 | 0.928 | 0.835 |

Notes: EPU, GPR, CPU, PSE, CSI, FX, and DR represent economic policy uncertainty, geopolitical risk, climate policy uncertainty, Arca Tech 100 Index, CSI 300 index, the exchange rate of US dollar to Chinese yuan, and a dummy variable for crisis periods, respectively. The “Total” column represents the OLS regression results of the variable on the total spillover effect, while the “Positive” and “Negative” columns respectively represent the regression results of the variable on positive and negative spillovers.
