# Supplementary material for: Asymmetric spillover connectedness between clean energy markets and industrial stock markets: How uncertainties affect it
Source: PLoS One. 2025 Mar 31;20(3):e0316171. doi: 10.1371/journal.pone.0316171 (PMC11957356; doi:10.1371/journal.pone.0316171)
Supplement: S1 File — Original data, code, and appendix content. (ZIP) [file pone.0316171.s001.zip › Supporting information/data and code/Raw data/Pearson correlation matrix.rtf]

HY	WP	SP	LC	OR	HC		
HY	1						
							
							
WP	0.533	1					
0							
							
SP	0.483	0.745	1				
0	0						
							
LC	0.649	0.781	0.764	1			
0	0	0					
							
OR	0.638	0.767	0.778	0.917	1		
0	0	0	0				
							
HC	0.676	0.765	0.759	0.936	0.877	1	
0	0	0	0	0			
