# Supplementary material for: Asymmetric spillover connectedness between clean energy markets and industrial stock markets: How uncertainties affect it
Source: PLoS One. 2025 Mar 31;20(3):e0316171. doi: 10.1371/journal.pone.0316171 (PMC11957356; doi:10.1371/journal.pone.0316171)
Supplement: S1 File — Original data, code, and appendix content. (ZIP) [file pone.0316171.s001.zip › Supporting information/data and code/Raw data/descriptive statistics.rtf]

Variable	Mean	SD	Skewness	Kurtosis	
HY	0.000500	0.0155	-0.752	13.97	
WP	0.000200	0.0226	-0.337	6.330	
SP	0.000700	0.0237	-0.358	5.941	
LC	0.000300	0.0167	-1.085	10.32	
OR	0.000400	0.0164	-0.935	9.764	
HC	0.000400	0.0176	-0.973	9.334	
